# Medial temporal ageing-related tau astrogliopathy below 66 years is associated with neurodegeneration

**DOI:** 10.1093/brain/awag011

**Published:** 2026-01-19

**Authors:** Sanne M M Vermorgen, Klara Gawor, Sandra O Tomé, Rik Vandenberghe, Christine A F von Arnim, Markus Otto, Philip Van Damme, Jochen H Weishaupt, Annemieke J M Rozemuller, Dietmar Rudolf Thal

**Affiliations:** Department of Pathology, Amsterdam UMC, Amsterdam 1081 HV, The Netherlands; Netherlands Brain Bank, Netherlands Institute for Neuroscience, Amsterdam 1105 BA, The Netherlands; Laboratory of Neuropathology, Department of Imaging and Pathology, and Leuven Brain Institute, KU Leuven, Leuven 3000, Belgium; Laboratory of Neuropathology, Department of Imaging and Pathology, and Leuven Brain Institute, KU Leuven, Leuven 3000, Belgium; Netherlands Brain Bank, Netherlands Institute for Neuroscience, Amsterdam 1105 BA, The Netherlands; Department of Neurology, University Hospitals Leuven, Leuven 3000, Belgium; Department of Neuroscience, KU Leuven, Leuven 3000, Belgium; Department of Neurology, Ulm University, Ulm 89081, Germany; Department of Geriatrics, University Medical Center Göttingen, Göttingen 37075, Germany; Department of Neurology, Ulm University, Ulm 89081, Germany; Department of Neurology, University of Halle, Halle 06120, Germany; Department of Neurology, University Hospitals Leuven, Leuven 3000, Belgium; Department of Neuroscience, KU Leuven, Leuven 3000, Belgium; Department of Neurology, Ulm University, Ulm 89081, Germany; Department of Pathology, Amsterdam UMC, Amsterdam 1081 HV, The Netherlands; Netherlands Brain Bank, Netherlands Institute for Neuroscience, Amsterdam 1105 BA, The Netherlands; Laboratory of Neuropathology, Department of Imaging and Pathology, and Leuven Brain Institute, KU Leuven, Leuven 3000, Belgium; Department of Pathology, UZ Leuven, Leuven 3000, Belgium

**Keywords:** ageing-related tau astrogliopathy, neurodegenerative disease, ageing, co-pathology

## Abstract

Ageing-related tau astrogliopathy (ARTAG) refers to aggregates of pathological tau protein in astroglial cells in the brain. Thorny astrocytes at the level of the glia limitans and/or white matter and granular/fuzzy astrocytes in the grey matter are characteristic for ARTAG, which is correlated with ageing. However, also rare cases with ARTAG below the age of 66 years have been reported.

We studied a cohort of 157 brains from donors deceased between 48 and 65 years of age received from the Leuven neuropathological research group and Netherlands Brain Bank in order to gain insight into ARTAG in the medial temporal lobe at younger age and to find underlying correlates that might be obscured by age-related co-pathologies in older cohorts. Analyses were also performed on two comparison cohorts (Leuven, 268 cases; and Netherlands, 397 cases), with ages ranging from 66 to 99 years.

Twenty-six of 157 cases (16.6%) between 48 and 65 years of age had ARTAG, mostly restricted to the medial temporal lobe. Only six cases exhibited ARTAG in lobar regions. ARTAG was found in all five previously described morphologies and locations: subpial, subependymal, perivascular, white matter and grey matter. In our young cohort, a significant correlation was found between ARTAG and the presence of neurodegenerative conditions of any kind and between ARTAG and age. When correcting for age and sex, the association between ARTAG and the presence of neurodegenerative conditions was upheld. There were no significant associations between ARTAG and specific proteinopathies, although trends were observed for α-synucleinopathy, tauopathy and TDP-43 proteinopathy diagnoses. The presence of lobar ARTAG was related to ARTAG severity in the young cohort. In the older cohorts, only age was significantly associated with ARTAG.

These results suggest a link between ARTAG in the medial temporal lobe of young individuals and pathological protein aggregation of any kind in the brain independent of age and might lead us to question whether ARTAG points to astrocytes as important players for selective vulnerability for the aggregation of pathological proteins in distinct brain regions in this patient population.

## Teaser

Vermorgen *et al.* examine tau aggregation in astrocytes in brains from middle-aged adults. They show that ARTAG is not restricted to elderly brains and is associated with neurodegenerative pathology, suggesting that astrocytes may contribute to regional vulnerability to protein aggregation in younger brains.

## Introduction

Hyperphosphorylated tau-positive thorn-shaped astrocytes in aged brains have been described since 2004.^1^ However, a harmonizing evaluation and terminology of the phenomenon of thorn-shaped and granular/fuzzy astrocytes was published only in 2016.^2^ Ageing-related tau astrogliopathy (ARTAG) refers to hyperphosphorylated tau (p-tau) aggregates in astrocytes, detectable by anti p-tauSer202/Thr205 or four repeat (4R)-tau immunohistochemistry.^3^ Five subtypes have been defined previously: subpial, subependymal, perivascular, white matter and grey matter ARTAG.^2^ In the grey matter, the astrocyte deposits take the form of granular/fuzzy astrocytes, whereas in the other locations, the afflicted astrocytes have a thorn-like shape. ARTAG is increasingly found with older age, leading to the definition of ARTAG as an age-related phenomenon.^2^

A few studies investigated clinicopathological associations and suggested associations between ARTAG and ventricular enlargement, basal atherosclerosis, brain atrophy, male sex, frontotemporal lobar degeneration with tau pathology (FTLD-tau),^4^ Alzheimer’s disease neuropathological changes,^5-7^ limbic-predominant age-related TDP-43 encephalopathy neuropathological changes (LATE-NC)^8-10^ and hippocampal sclerosis.^11^ Some studies also found associations between clinical symptoms, such as cognitive impairment,^12^ worsening language and visuospatial functions^3^ and dementia,^13^ although others failed to find an association with cognitive decline.^5,14^ However, the strong association with age, which is, in itself, associated with pathological brain changes and cognitive decline, might make these associations difficult to interpret.

In very rare cases, ARTAG has been reported in younger people.^1,15^ The presence of ARTAG in young individuals cannot be explained simply by age and could be indicative of underlying pathogenic mechanisms associated with ARTAG, which are overshadowed by age in older cohorts and as shown in the case report of a 58-year-old with co-morbid prion disease.^15^

To understand ARTAG better and to elucidate potential clinical or neuropathological associations, we studied a younger cohort with an age range of 48–65 years. These associations might clarify broader pathogenic mechanisms related to pathological protein misfolding.

## Materials and methods

### Database selection

In order to investigate ARTAG in younger individuals, the Netherlands Brain Bank (NBB, Amsterdam, the Netherlands, https://www.brainbank.nl) and the KU Leuven Brain collection (KUL) were searched for brain autopsies from individuals below the age of 66 years (48–65 cohort), because 65 years is often considered the threshold for onset of ageing-related conditions. Given that no cases in both databases showed ARTAG below the age of 48 years, we used 48 years as the lower threshold for our cohort. Case selection criteria were as follows: (i) age range from 48 to 54 years; and (ii) case collection date between 1 January 2016 and 31 December 2022 (for NBB cases only; for KUL, all cases were included regardless of collection date).

For comparison, we searched both KUL and NBB databases for brain autopsies from individuals between 66 and 99 years of age (66+ cohort), collected in the same timespan (for NBB, 1 January 2016–31 December 2022; for KUL, no selection on collection date), where ARTAG was assessed (present or absent) by the neuropathologist at the time of diagnosis or assessed afterwards (for the KUL cases).

No further case selection was done in the 48–65 cohort or in the 66+ cohort, to preserve a diverse and natural dataset. All autopsies were performed in accordance with Dutch, Belgian and German law after approval by respective ethical committees (the Netherlands: 2009/148; Belgium: UZ/KU Leuven: S-59292, S-52791, S-65097 and S-55312; Germany: Ulm University: 54/08). The data collection for scientific purposes was approved by the local ethical committees (KU/UZ Leuven, S-59295 and S-65147; NBB, 2009/148). All NBB and KUL donors gave informed consent for the use of their tissue and clinical data for scientific research.

### Cohort descriptions

**Figure 1:**
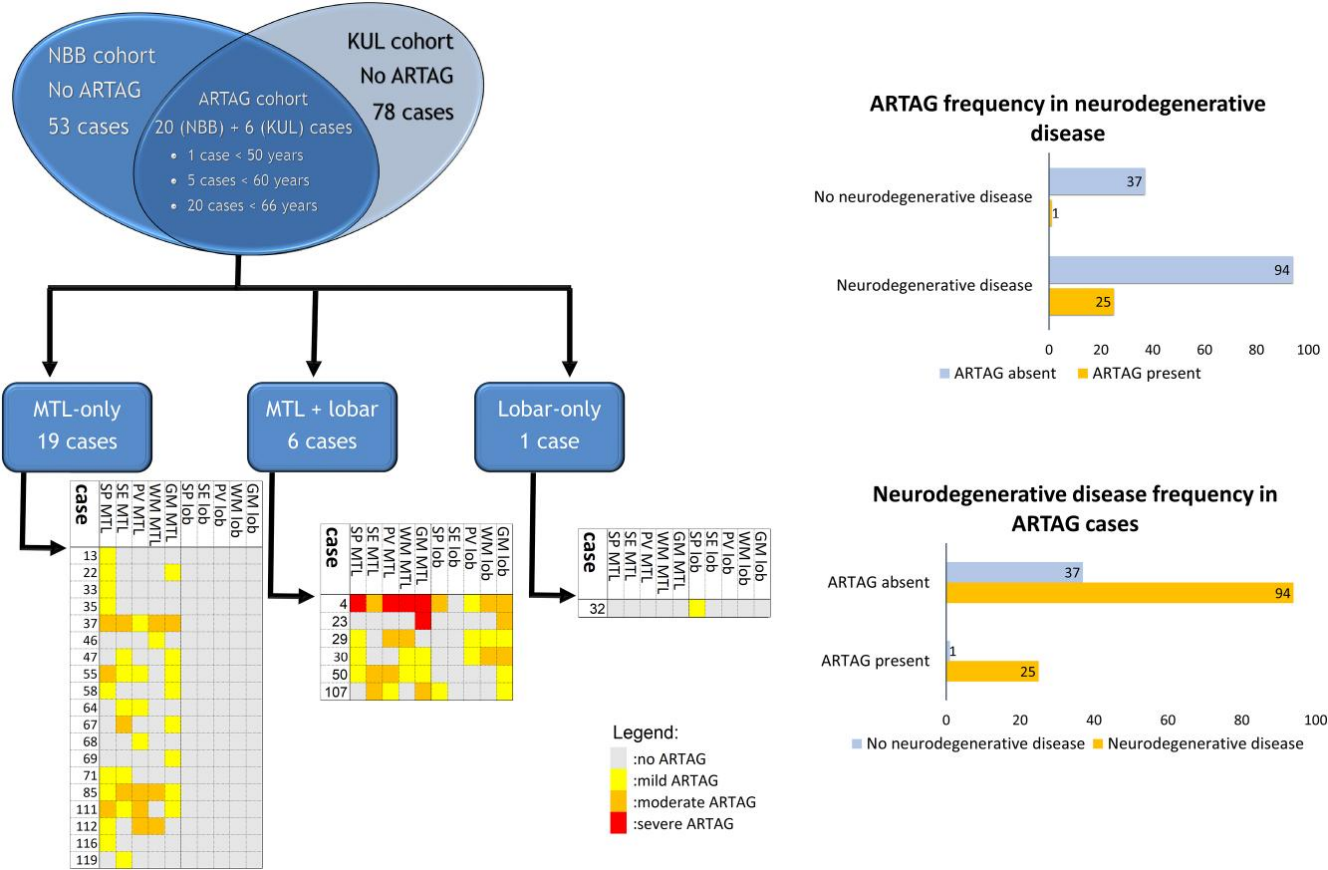
**Overview of the young cohort.** Overview of the combined 48–65 cohort with summary of ARTAG distribution, ARTAG frequency in relationship to neurodegenerative disease, ARTAG type and ARTAG severity (no ARTAG, mild ARTAG, moderate ARTAG or severe ARTAG). ARTAG = ageing-related tau astrogliopathy; GM lob = grey matter ARTAG in the lobar area; GM MTL = grey matter ARTAG in the medial temporal lobe; PV lob = perivascular ARTAG in the lobar area; PV MTL = perivascular ARTAG in the medial temporal lobe; SE lob = subependymal ARTAG in the lobar area; SE MTL = subependymal ARTAG in the medial temporal lobe; SP lob = subpial ARTAG in the lobar area; SP MTL = subpial ARTAG in the medial temporal lobe; WM lob = white matter ARTAG in the lobar area; WM MTL = white matter ARTAG in the medial temporal lobe.

**Figure 2:**
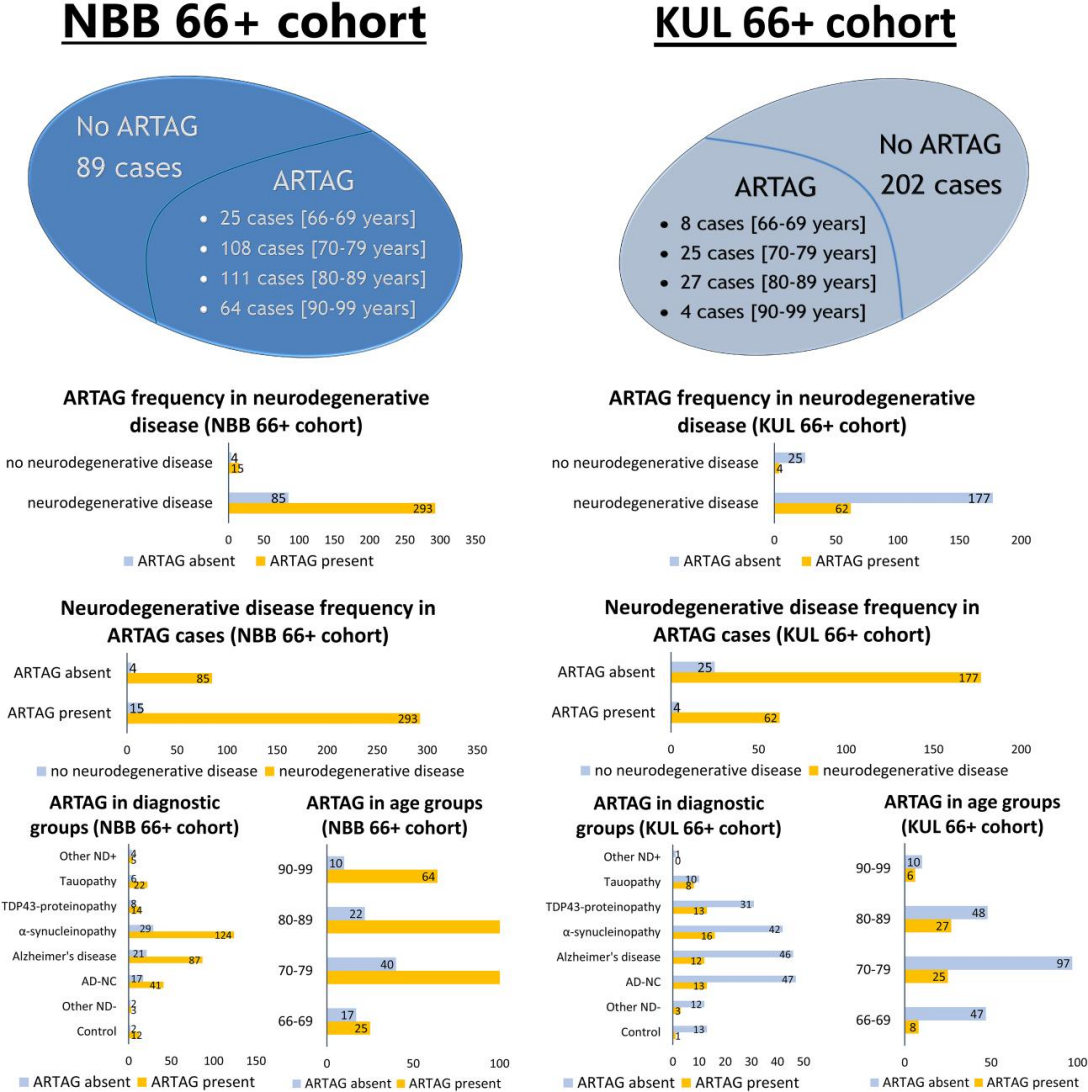
**Overview of the 66**+ **cohorts.** Netherlands Brain Bank (NBB) on the *left* and KU Leuven Brain collection (KUL) on the *right*. The number of cases with represented ARTAG are graphically according to presence of neurodegenerative disease, age and diagnostic category. AD-NC = Alzheimer's disease neuropathological changes AD-NC; Alzheimer's disease neuropathological changes ARTAG = ageing-related tau astrogliopathy; ND = neurodegenerative changes.

See Figs 1 and 2 for a visual representation of the 48–65 cohort and both 66+ cohorts. For the 48–65 cohort, a total of 157 cases were selected, of which 73 cases belonged to the NBB dataset and 84 cases came from the KUL dataset. Age ranged from 48 to 65 years [mean 59.48 years, standard deviation (SD) 4.45 years]. Details about tissue preparation can be found in previous publications for the KUL^16^ and the NBB^17^ databases and are described in the Supplementary material, ‘Methods’ section. Briefly, fresh brain tissue was fixed for 4–6 weeks in 4% formalin, before imbedding in paraffin and sectioning in 4–6 µm slices. Antibody information can be found in Supplementary Table 1. All cases were evaluated using light microscopy by two neuropathologists (for NBB cases, S.V. and A.J.M.R.; for KUL cases, S.V. and D.R.T.). Neuropathological diagnoses were initially categorized in seven groups (for inclusion and exclusion criteria, see Table 1). Then further pooling of categories was done, resulting in two categories based on the presence or absence of neurodegenerative changes (ND): ND− (Control and Other ND−) versus ND+ (AD-NC, AD, Tau, TDP-43, α-Synuclein and Other ND+).

**Table 1:** Description and criteria of the seven diagnostic categories

Diagnostic category	Description
Control	Absence of α-synucleinopathy or TDP-43 proteinopathy Amyloid plaque phase 0 Braak-NFT stage 0–2 Absence of diagnoses described in category Other ND−
AD-NC	Absence of α-synucleinopathy or TDP-43 proteinopathy Braak-NFT stage 3 or higher Amyloid plaque phase 1 or higher Clinically non demented
AD	Braak-NFT stage 3 or higher and amyloid plaque phase 3 or higher Clinically demented α-Synucleinopathy absent or restricted to the amygdala TDP-43 proteinopathy absent or consistent with limbic predominant age-related TDP-43 encephalopathy (LATE-NC)
α-Synuclein	α-Synucleinopathy (excluding amygdala-only when case fits category AD, as described above) Any amyloid plaque phase Any Braak-NFT stage Absence of TDP-43 proteinopathy
TDP-43	TDP-43 proteinopathy in any confirmation (FTLD-TDP, ALS or LATE) In case of LATE-NC: Braak-NFT stage and amyloid plaque phase insufficient for diagnostic category AD as described above
Tau	Primary tauopathy (Progressive supranuclear palsy, corticobasal degeneration, Pick’s disease, hereditary tauopathy) Primary age-related tauopathy and argyrophilic grain disease were included in the AD-NC category instead of the tau category
Other ND+	Neurodegenerative disease not fitting in the above categories. Nine cases: three cases with FTLD-FET; three cases with TDP-43-negative ALS; one case with Friedreich’s ataxia; one case with spinocerebellar ataxia 6; one case with Huntington’s disease
Other ND−	Other non-neurodegenerative diseases and diagnoses. Consists of five types of brain abnormalities: Malignancy (four cases) Neuro-inflammation (nine cases) Vascular or ischaemic damage (seven cases) Trauma (one case) Developmental disorder (one case)

AD = Alzheimer's disease; AD-NC = Alzheimer's disease neuropathological changes; ALS = amyotrophic lateral sclerosis; ARTAG = ageing-related tau astrogliopathy; Braak NFT = Braak staging for neurofibrillary tangles; FTLD-FET = frontotemporal lobar degeneration with FET inclusions; FTLD-TDP = frontotemporal lobar degeneration with TDP inclusions; ND = neurodegenerative changes; LATE = limbic predominant age-related TDP-43 encephalopathy; TDP-43 = TAR DNA-binding protein 43.

The least-represented groups in the 48–65 cohort were Other ND+ (9 of 157 cases), Tau (12 of 157 cases) and α-Synuclein (15 of 157 cases). The category Control consisted of 18 of 157 cases (11.5%). See Table 2 for mean age, mean brain weight, median clinical dementia rating score and percentage male sex for each of the seven diagnostic groups. Descriptive statistics for the other variables and graphical representation of neuropathological features can be found in Supplementary Table 2 and Supplementary Fig. 1. There was no significant difference in age or brain weight between the diagnostic groups.

**Table 2:** Group characteristics of the 48–65 cohort

Diagnostic category	*n*	% Male	Age, years; mean (SD)	Brain weight, g; mean (SD)	Median Clinical Dementia Rating
Control	18	38.9%	58.39 (5.10)	1221.35 (157.13)	0
Other ND−	20	25%	58.00 (4.15)	1230.48 (215.14)	0
AD-NC	28	57.1%	59.03 (4.87)	1300.11 (152.79)	0
AD	22	63.6%	60.82 (3.19)	1178.55 (186.14)	3
α-Syn	15	66.7%	60.00 (5.13)	1344.00 (112.90)	0.5
TDP-43	33	63.6%	60.48 (4.43)	1183.77 (219.34)	2
Tau	12	66.7%	61.67 (2.99)	1268.42 (174.09)	2
Other ND+	9	88.9%	56.33 (4.87)	1191.56 (152.79)	0

The categories above the dotted line form the pooled category ND−. The categories below the dotted line form the pooled category ND+. α-syn = α-synuclein; AD = Alzheimer's disease; NC = neuropathological changes; ND = neurodegenerative changes.

The 66+ cohort consisted of 397 cases from the NBB dataset and 268 cases from the KUL, giving a total of 665 cases. The NBB dataset consisted mainly of cases in the α-Synuclein and AD diagnostic groups (38.5% and 27.2%, respectively), whereas the Control, Other ND− and Other ND+ groups were smallest (3.5%, 1.3% and 2.3%, respectively). For the KUL dataset, the majority of cases belonged to the AD-NC, α-Synuclein and AD groups (22.4%, 21.6% and 21.6%, respectively), whereas Control, Other ND− and Other ND+ categories also showed the lowest percentages of cases (5.2%, 5.6% and 0.4%, respectively).

### Variables

Several clinical and neuropathological variables were assessed (Table 3). Evaluation of the presence, location and severity of ARTAG (in a three-tiered scale: 1, occasional; 2, focally accentuated; or 3, widespread) was done for the 48–65 cohort according to the consensus guidelines,^2^ using anti-phosphorylated tau [anti-pTau (S202/T205) mouse antibody AT8, Pierce Biotechnology]-stained slides of medial temporal lobe (MTL; regions including amygdala and hippocampus) and lobar regions [occipital (all cases), parietal, lateral temporal and frontal (NBB cases only) cortex]. For six cases, brainstem (medulla oblongata and pons) and subcortical (thalamus and striatum) AT8-stained slides were available. Owing to small sample size, analyses were done only on MTL ARTAG, without subgrouping in the different subtypes. ARTAG severity was rated for general presence of ARTAG, regardless of ARTAG subtype.

**Table 3:** List of evaluated variables

Variable	Name	Description	Staging system reference
Age	Age	Age at time of death	–
Sex	Sex	Biologically male or female	–
Neurofibrillary tangles (NFT)	Braak-NFT	Staging of neurofibrillary tangle pathology (6 stages) according to the consensus of the BrainNet Europe Consortium	Alafuzoff *et al*.^18^
Amyloid plaques	Aβ phase	Phases of β-amyloid deposition (five phases)	Thal *et al*.^19^
Amyloid score (A)	A-score	National Institute on Aging–Alzheimer’s Association guidelines for the assessment of amyloid plaques (three tiers)	Montine *et al*.^20^
NFT score (B)	B-score	National Institute on Aging–Alzheimer’s Association guidelines for the assessment of neurofibrillary tangle score (three tiers)	Montine *et al*.^20^
Neuritic plaque score (C)	C-score	CERAD neuritic plaque score (three tiers)	Montine *et al*.,^20^ Mirra *et al*.^21^
Cerebral amyloid angiopathy (presence)	CAA	Presence of β-amyloid deposition in vessel walls	–
Cerebral amyloid angiopathy (type)	CAA-type	Type of cerebral amyloid angiopathy (type 1 or type 2)	Thal *et al*.^22^
Cerebral amyloid angiopathy (stage)	CAA-stage	Staging of cerebral amyloid angiopathy (three stages)	Thal *et al*.^23^
α-Synuclein (presence)	αSyn	Presence of immunoreactivity for anti-α-synuclein antibody (in the form of Lewy bodies, Lewy threads or Papp–Lantos bodies)	–
Lewy bodies	Braak-LBD	Staging of Lewy body pathology (six stages)	Braak *et al*.^24^
TDP-43 pathology (presence)	TDP-43	Presence of immunoreactivity for anti-pTDP-43 antibody (in the form of neuronal cytoplasmic inclusions, neuronal intranuclear inclusions or threads)	–
(Micro)infarcts	Infarct	Presence of ischaemic brain damage in the form of infarcts or micro-infarcts	–
Brain weight	Weight	Brain weight (fresh) at autopsy	–

Column 1, short description of variable; column 2, short name of variable; column 3, antibody used in the evaluation; and column 4, reference to staging system. Supplementary Table 1 for antibodies used. Description column indicates antibody used in the evaluation. Antibodies used are listed in Supplementary Table 1.

For one case, no α-synuclein stain was available, and brain weight was unknown for three cases. Clinical dementia rating scale was not included in our analysis, owing to too many missing values (46 cases).

For the variables α-synuclein (presence) and TDP-43 pathology (presence), we chose to pool all subtypes of the respective pathological proteins, despite the molecular differences in misfolded protein in these subtypes and clinical variability, to reduce variables with a small number of positive cases. Tentative analysis of these subtypes showed no significant differences amongst groups. See Supplementary Figs 2 and 3 for graphical representation of the TDP subtypes with results of this tentative analysis.

### Statistical analysis

Statistical analysis was performed using IBM SPSS Statistics version 29. Associations between variables were first investigated with Pearson’s χ^2^ test for categorical variables and the Mann–Whitney U-test non-parametrical comparison of distributions for ordered variables, with Fisher exact correction (two-sided) when necessary. Significant variables (*P* < 0.05) were included in a logistic regression model to investigate the associations while controlling for age, sex and other covariates.

### Comparison between KUL and NBB datasets

Several significant differences were found between the patient cohorts from KUL and NBB (Supplementary Table 3). NBB cases showed more α-synuclein, amyloid (both plaques and angiopathy) and neuritic plaque pathology, whereas KUL cases more frequently had TDP-43 pathology (specifically, more amyotrophic lateral sclerosis cases). NBB cases were older and had higher clinical dementia rating scores. There were more ARTAG cases with a clinical dementia rating in the NBB dataset, but this difference remained statistically insignificant.

However, there was a significant difference in the prevalence of ARTAG between the KUL and NBB datasets in the 66+ cohort (77.8% in the NBB dataset versus 24.6% in the KUL dataset; χ^2^ = 182.304, *P* < 0.001).

## Results

### 48–65 cohort

**Figure 3:**
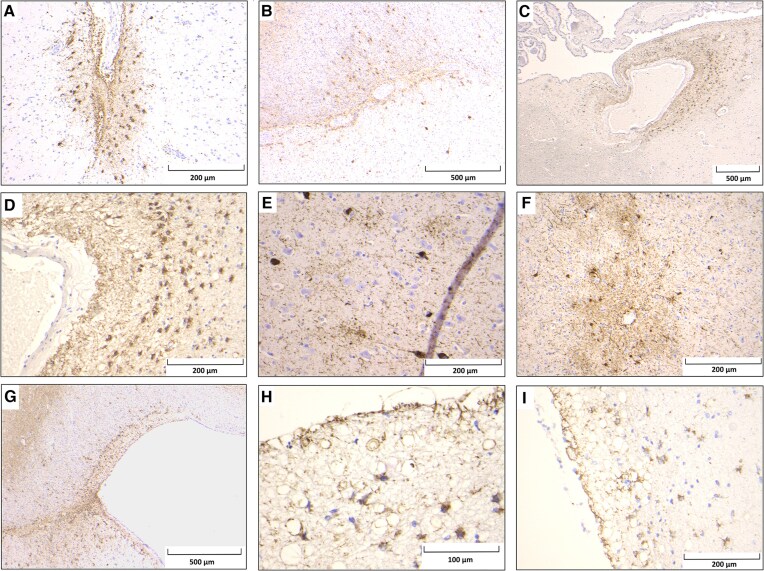
**ARTAG examples.** (**A**–**C**) Perivascular ‘thorny’ ARTAG, spreading out into the white matter. (**D**) Higher magnification of perivascular ARTAG in **C**. (**E** and **F**) ‘Fuzzy’ grey matter ARTAG. (**G**) Subependymal ‘thorny’ ARTAG. (**H** and **I**) Subpial ‘thorny’ ARTAG. ARTAG = ageing-related tau astrogliopathy.

The datasets of the KUL and NBB 48- to 65-year-old cohorts (48–65 cohort) were pooled for the analysis of differences between cases with and without ARTAG (see Figs 1 and 2 for an overview of the cohorts and Fig. 3 for examples of ARTAG). Briefly, ARTAG was present in 16.6% of cases (26 of 157). ARTAG severity was mostly low: 53.8% of ARTAG cases had occasional ARTAG, 38.5% had focally accentuated ARTAG and 7.7% had widespread ARTAG. ARTAG was present in all previously described subtypes (subpial, subependymal, perivascular, white matter and grey matter). ARTAG was mostly present in the MTL (MTL-ARTAG), with only six cases having additional presence of ARTAG in the lobar sections. One case had only a few thorny astrocytes subpially, without any ARTAG in the MTL. This case did not meet the criteria for chronic traumatic encephalopathy and was thus included as lobar-only ARTAG. From the six ARTAG cases with stained sections of the brainstem and subcortical grey matter, no cases showed ARTAG in brainstem or subcortical regions.

**Figure 4:**
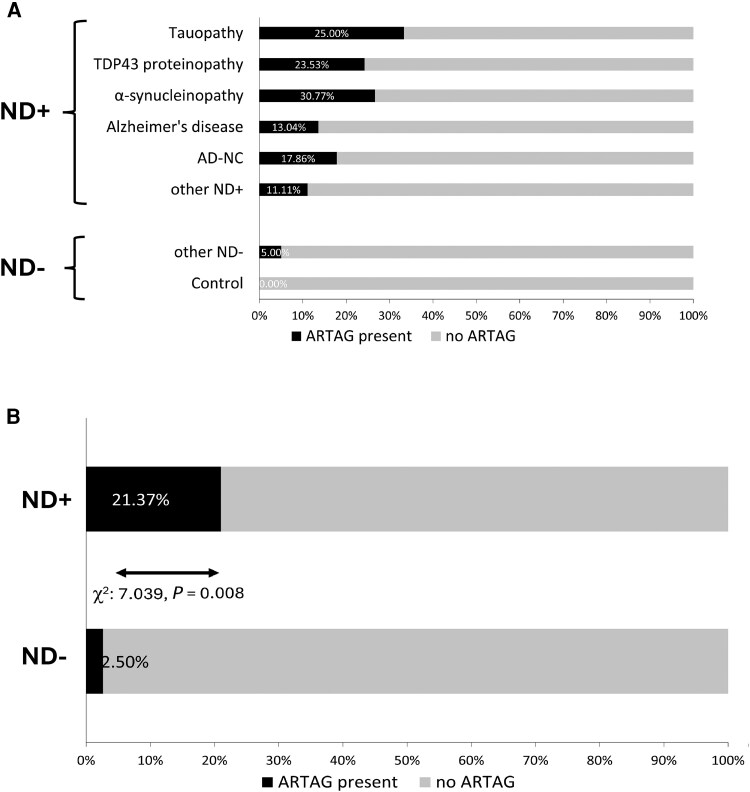
**ARTAG in diagnostic groups.** (**A**) Percentage of cases with ARTAG, according to diagnostic category. (**B**) ARTAG in pooled diagnostic groups (ND+ or ND−). Percentage of ARTAG in the pooled diagnostic categories ND+ versus ND− (i.e. presence versus absence of neurodegenerative changes). Significance level is displayed by a double-headed arrow between the bars. The displayed percentages represent the fraction within each group with ARTAG present: 21.55% (25 cases) in the ND+ group versus 2.44% (1 case) in the ND− group. ARTAG = ageing-related tau astrogliopathy; AD-NC = Alzheimer's disease neuropathological changes; ND = neurodegenerative changes. = = = ADAlzheimer's disease; NCneuropathological change; NDneurodegeneration

Of the 26 cases with ARTAG in the 48–65 cohort, 25 cases belonged to the pooled category ND+, the category with neurodegenerative changes. The only case with ARTAG that did not belong to this ND+ category was a case that belonged in the category ‘other ND−’. This case had a diagnosis of Aicardi syndrome, a neurodevelopmental disorder. See Fig. 4A for the distribution of ARTAG in the seven diagnostic categories. Cases in the ND+ category were more often male than in the ND− category (63.8% versus 36.6%; χ^2^ = 9.133, *P* = 0.003). No significant differences were found in age and brain weight between the ND+ and ND− categories.

**Figure 5:**
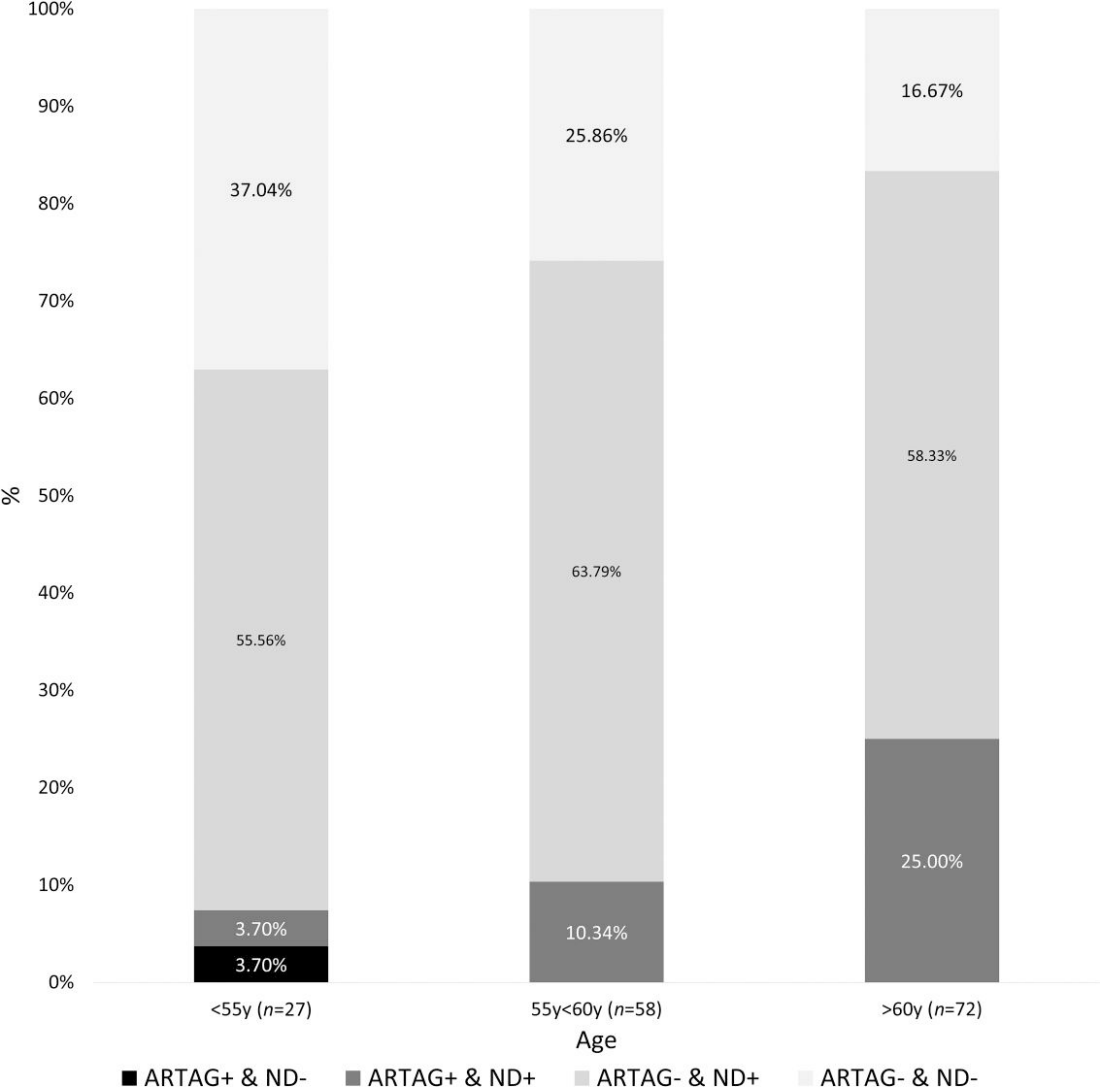
**Prevalence of ARTAG and neurodegenerative changes (ND+ or ND−).** Prevalence of ARTAG in the pooled categories ND+ and ND−, divided into three age groups. The black bar represents cases with ARTAG and without neurodegenerative changes (ARTAG+ & ND−). The dark grey bar represents the cases with ARTAG, and presence of neurodegenerative changes (ARTAG+ & ND+). The lighter grey represents the cases without ARTAG and presence of neurodegenerative changes (ARTAG− & ND+). The lightest grey bar represents cases without ARTAG and without neurodegenerative changes (ARTAG− & ND−). The *x*-axis represents the age groups (55 years or younger; between 55 years and 60 years; and older than 60 years) and the *y*-axis represents the cumulative percentages within each age group. Presence of neurodegenerative changes and presence of ARTAG increase with age. The group ARTAG+ & ND− is limited to a single case: this is a case with a neurodevelopmental disorder (Aicardi syndrome). ARTAG = ageing-related tau astrogliopathy.

Univariate analysis (by non-parametric χ^2^ and Mann–Whitney U-tests) showed a significant association between presence of ARTAG and age at death (Mann–Whitney U = 2208.5, *P* = 0.017), and between presence of ARTAG and the pooled category (ND+) (χ^2^ = 7.039, *P* = 0.008; see Figs 4B and 5). No significant differences were found for the other tested variables [sex, brain weight or presence of (micro)infarcts], and there was no association with the amount of different pathological proteins present (multiproteinopathy). None of the pathological protein variables (Braak-NFT stage, Aβ phase, A-score, B-score, C-score, CAA, CAA-type, CAA-stage, α-synuclein, Braak-LB and TDP-43) showed significant associations with ARTAG, but a trend was seen between the presence of ARTAG and the diagnostic categories ‘α-Synuclein’, ‘TDP-43’ and ‘Tau’ (Fig. 4A).

When performing logistic regression with age, sex and ND+ as independent variables and with the presence of ARTAG as a dependent variable, the association between ARTAG and ND+ remained significant [coefficient = 0.103 (0.013–0.815), *P* = 0.031], whereas the association between ARTAG and age lost significance [coefficient = 1.107 (0.984–1.246), *P* = 0.091]. Sex was not significantly associated with ARTAG. When adding the dataset location (NBB versus KUL) as a dependent variable to control for dataset-specific variance, ND+ remained significantly associated with ARTAG [coefficient *=* 0.119 (0.015–0.949), *P* = 0.044], whereas age was again not significantly associated [coefficient = 1.067 (0.944–1.205), *P* = 0.298].

In the ARTAG group of the 48–65 cohort, presence of lobar ARTAG (*n* = 6 cases) was associated with ARTAG severity (U = 108.5, *P* = 0.013) and weakly with B-score (U = 101.5, *P* = 0.041), whereas there was no association between ARTAG and Braak-NFT score (U = 96, *P* = 0.094). Logistic regression with ARTAG severity and B-score as dependent variables and with lobar ARTAG as an independent variable showed a significant association with ARTAG severity only [ARTAG severity: coefficient = 12.366 (1.144–133.679), *P =* 0.038; B-score: coefficient = 4.654 (0.901–24.050), *P* = 0.066]. The marginal statistical significance of the association between B-score and ARTAG, the absence of an association between Braak-NFT stage and ARTAG and the disappearance of significance of B-score when including ARTAG severity in the multivariate analysis indicate that the association between B-score and ARTAG is most likely not to be clinically significant.

ARTAG severity was not associated with age, Braak-NFT stage, Braak-LB stage, Aβ phase, brain weight and multiproteinopathy (Supplementary Table 4A).

### 66+ cohort

The cohorts of cases with an age >65 years (66+ cohorts) showed a different pattern of associations. Because of the significant difference in ARTAG prevalence (see Materials and Methods section) between the NBB and KUL datasets in the 66+ comparison cohort, we ran separate analyses for NBB and KUL. In the NBB 66+ cohort, 308 of 397 cases (77.8%) had ARTAG. For this cohort, the prevalence of cases in the ND− group was low (19 cases), of which 15 cases had ARTAG (78.9%). Of these 15 cases with ARTAG and ND−, seven cases had Braak-NFT stage 2, seven cases had Braak-NFT stage 1 and one case had Braak-NFT stage 0. The single ARTAG+ ND− case who had a Braak-NFT stage of 0 had a history of pituitary adenoma resection. Of the four ARTAG-negative cases in the ND− group of the NBB 66+ cohort, two cases had Braak-NFT stage 0 and two cases had Braak-NFT stage 1. In the KUL 66+ cohort, 66 of 268 cases (24.6%) had ARTAG. The prevalence of ND− in the KUL 66+ cohort was 29 cases, of which four cases had ARTAG (13.8%). For the KUL 66+ cohort in the ND− group without ARTAG, five cases had Braak-NFT stage 2, 17 had Braak-NFT stage 1 and three had Braak-NFT stage 0, while three of four cases in the ND− group with ARTAG had Braak-NFT stage 2 and the fourth case had Braak-NFT stage 1. Accordingly, all cases with ARTAG in the 66+ cohort had at least low levels of primary age-related tauopathy as initial neurodegenerative changes, except for a single case. Univariate analysis found a significant association only between ARTAG and age (NBB: U = 17159.5, *P* < 0.001; KUL: U = 8570, *P* < 0.001). No significant effects were found between ARTAG and ND+, sex, brain weight, presence of (micro)infarcts, Braak-NFT stage, Aβ phase, CAA, α-synuclein or TDP-43 (Supplementary Table 4B). In logistic regression models with age, ND+ and sex as independent variables and with ARTAG as a dependent variable, only age was significant [NBB: coefficient = 1.062 (1.030–1.094), *P* < 0.001; KUL: coefficient = 1.064 (1.025–1.104), *P* = 0.001].

## Discussion

Studies investigating neuropathological changes in young cohorts are rare. Yet, these cohorts are important because they might elucidate pathophysiological associations that are clouded by age-related phenomena in older cohorts. In our study of a cohort of cases below the age of 66 years, ARTAG was found in 16.6% of cases, indicating that ARTAG is not restricted to elderly brains. We found an association between MTL ARTAG and pathological protein aggregation in the brain, in a protein-independent way. This association could not be explained by age at death or sex of the affected individuals. No significant associations were found between specific protein-aggregating diseases or with specific neuropathological features, although trends were seen towards more ARTAG in α-synucleinopathies, primary tauopathies and TDP-43 proteinopathies.

To compare our results in younger brains with those observed in older individuals in the KUL and NBB Brain Banks, we repeated our analysis on two cohorts of brains from older individuals (from 66 to 99 years of age). Owing to the significant difference in ARTAG prevalence between the KUL and NBB cohorts, not seen in the young (48–65) cohort, we analysed the 66+ brains in two separate cohorts (KUL and NBB). In both 66+ cohorts, age was the only parameter associated with ARTAG. An association between pathological protein aggregation and ARTAG was not observed in these older cohorts. Potential explanations for the different associations in individuals 48–65 years old and individuals >65 years old could be: (i) that neurodegenerative pathologies, or at least low primary age-related tauopathy (Braak-NFT stages 1–2), are too prevalent at >65 years of age to see an association of the prevalence of abnormal protein accumulation with ARTAG; (ii) that pathogenic differences exist in ARTAG of younger patients compared with older ones; or (iii) that young people with neurodegenerative disease are distinct from their older counterparts, which might be attributable to differences in the aetiology of neurodegenerative disease.^25^ Cases who fitted the ND− category in our study were rare in our 66+ cohorts. No association with Braak stage tau could be found, but there appeared to be a trend towards higher Braak tau stage in the ARTAG-positive cases in the ND− cases in both the NBB and KUL 66+ cohorts. As stated earlier, brain ageing is associated with increased risk for neurodegenerative disease,^26,27^ which is reflected by the decreasing prevalence of control brains, without any neurodegenerative changes in our cohorts: 3.5% and 5.2% in our two aged cohorts, compared with 11.5% in the younger cohort.

Studies focusing on the association between ARTAG and several proteinopathies have shown conflicting results. Although several studies have pointed towards an association between ARTAG and several neuropathological changes,^4-11,28^ the evidence for an association between ARTAG and cognitive decline is contradictory.^5,12-14,29,30^ These studies have, however, mainly focused on aged cohorts, because both ARTAG and pathological protein aggregation in the course of neurodegenerative disease are more prevalent in older populations. However, ARTAG is not limited to aged individuals, as is reported in some studies in sporadic Creutzfeldt–Jakob disease with a younger disease population.^15,31^ The association between ARTAG and pathological protein aggregation found in our young cohort might suggest a link between hyperphosphorylated tau aggregation in astrocytes and vulnerability to neurodegenerative disease, especially in younger individuals. ARTAG might be a symptom of an underlying vulnerability for pathological protein aggregation and subsequent neurodegenerative disease.

Interestingly, the only case in our young cohort who had ARTAG but did not have neurodegenerative changes in the brain was a case with the congenital neuroinflammatory Aicardi syndrome.^32^ Pathological tau aggregation has been described in neurodevelopmental disorders, even at a very young age.^33,34^ Given that neuroinflammatory events are well known to occur also in neurodegenerative diseases,^35-38^ even in asymptomatic stages,^39^ it cannot be excluded that neuroinflammatory events are involved in the pathogenesis of ARTAG. Future studies are required to address this question.

Astrocytes, especially at the glia limitans, have, amongst many other functions, a vital role in the blood–brain and blood–CSF barriers, with an important role in brain homeostasis, through the control of the passage of molecules, solutes and immune cells, including proteins that accumulate in neurodegenerative diseases.^40-42^ Blood–brain barrier dysfunction has been hypothesized as an underlying mechanism of neurodegeneration.^43^ Given that ARTAG is highly present at these important barrier sites, it could be hypothesized that ARTAG is a sign or symptom of glia limitans dysfunction instead of simply being a ‘harmless’ ageing-related phenomenon. Alternatively, ARTAG might be a sign of astrocyte senescence and accelerated brain ageing in individuals with neurodegenerative disease.

More functional studies of glial cells with pathological tau aggregation could lead to fundamental knowledge of neurodegenerative disease mechanisms. Thorn-shaped astrocytes appear to develop in senescent astrocytes in a context of increased protein phosphorylation, with upregulation of several kinases with the potential to phosphorylate a large number of substrates.^44^ This increased phosphorylation rate could explain the fact that ARTAG is more frequent not only in tauopathies, but also in other protein-aggregating diseases. Also connexin-43 and aquaporin-4, two important astrocytic proteins with roles in the blood–brain barrier and brain–CSF interface, were found to be increased in ARTAG.^45^ Waste-clearing pathways, probably including aquaporin-4-dependent pathways, were recently proposed,^46^ which might link ARTAG to deficient waste clearing.

As described earlier, the MTL is the hotspot for ARTAG.^1,2,4,47^ This region, especially the amygdala, is also a predilection site for other proteinopathies, such as AD,^19,48^ LATE-NC^49^ and amygdala-predominant Lewy body disease,^50,51^ frequently occurring together with pTau in a subgroup of AD cases that shows colocalization of pTau, TDP-43 and α-synuclein within the same neurons.^52^ Comorbidity and multiproteinopathy in neurodegenerative diseases are very common,^27,53-55^ indicating crosstalk and interactions between the different pathological proteins, especially in regions where diseases overlap, such as the MTL.^56,57^ More research is necessary to elucidate the mechanisms of these tendencies for multiproteinopathy in the MTL. Studies focusing on the putative selective vulnerability of the MTL to neurodegeneration could lead to further understanding of the underlying pathophysiology of different neurodegenerative diseases and their link with ARTAG.

This study has a few limitations. Our cohort of younger individuals with ARTAG is small, and the number of control cases is also low, which resulted in low statistical power. We combined Brain Bank cases from Leuven and the Netherlands in order to enlarge our dataset, while controlling for the potential confounding effect of differences in the two dataset Brain Bank populations by adding database origin as a covariate in our analysis. In our 48- to 65-year-old cohort, there was no difference in prevalence of ARTAG between KUL and NBB datasets, but in our 66+ cohort there was a significant difference, indicating that caution is warranted when combining datasets from different tissue resources, probably owing to different recruitment strategies. Future studies with larger sample sizes and community-based samples are required to explore and corroborate the associations we found in greater detail.

The low prevalence of ARTAG in cases younger than 66 years also made it impossible to analyse the different subtypes of ARTAG, or the ARTAG constellations as described by Kovacs *et al*.^4^ in 2017 in older individuals. Moreover, the differing pathways found for each ARTAG subtype, described by Kovacs *et al*.,^47^ could not be studied in our cohort in detail.

## Conclusion

In conclusion, we found an association between MTL-ARTAG and: (i) age; and (ii) neurodegenerative changes in a 48- to 65-year-old cohort. The association between MTL-ARTAG and neurodegenerative changes remained significant when controlling for age. In our 66+ cohorts, only an association between ARTAG and age was found, without demonstrable associations between ARTAG and neurodegenerative changes. Given that all ARTAG cases in the 66+ ND− group exhibited at least Braak-NFT stage 1, except for a single case, it is tempting to speculate that ARTAG is triggered by an underlying vulnerability to the neurodegenerative process in younger individuals. Neuroinflammation might play an important role in this process. Accordingly, ARTAG in the MTL in young individuals is not merely an age-related phenomenon, but a disease-associated or -triggered phenomenon. This points to an important role of astrocytes in the development of neurodegenerative diseases in young individuals, which calls into question whether astrocytic alterations in a given brain region contribute to the concept of selective vulnerability. Our finding that in young individuals ARTAG is mainly found in the MTL and is associated with neurodegeneration in general would support this idea.

## Supplementary Material

awag011_Supplementary_Data

## Data Availability

The data that support the findings of this study are available from the corresponding author, upon reasonable request.
