# Correction to: Medial temporal ageing-related tau astrogliopathy below 66 years is associated with neurodegeneration

**DOI:** 10.1093/brain/awag255

**Published:** 2026-08-03

Sanne M. M. Vermorgen, Klara Gawor, Sandra O. Tomé, Rik Vandenberghe, Christine A. F. von Arnim, Markus Otto, Philip Van Damme, Jochen H. Weishaupt, Annemieke J. M. Rozemuller, Dietmar Rudolf Thal, Netherlands Brain Bank. Medial temporal ageing-related tau astrogliopathy below 66 years is associated with neurodegeneration. *Brain*. 2026;149:2791–2802. https://doi.org/10.1093/brain/awag011

In the originally published version of this manuscript, there were errors in the author affiliations.

These errors have been corrected, and all authors have been assigned the correct associated affiliation.

