# Supplementary material for: Medial temporal ageing-related tau astrogliopathy below 66 years is associated with neurodegeneration
Source: Brain. 2026 Jan 19;149(8):2791–802. doi: 10.1093/brain/awag011 (PMC13431666; doi:10.1093/brain/awag011)

Supplementary information for:

Mesiotemporal aging-related tau astrogliopathy below 66 years is associated with neurodegeneration

Sanne M. M. Vermorgen[*1,2], Klara Gawor[3], Sandra O. Tomé[3], Netherlands Brain Bank[2], Rik Vandenberghe[4], Christine A. F. von Arnim[4,5], Markus Otto[4,6], Philip Van Damme[4], Jochem H. Weishaupt[4], Annemieke J.M. Rozemuller[1,2, †], Dietmar Rudolf Thal[3,7, †]

[†]These authors contributed equally to this work.

Author affiliations:

1: Department of Pathology, Amsterdam UMC, 1081 HV, Amsterdam, The Netherlands

2: Netherlands Brain Bank, Netherlands Institute for Neuroscience, 1105 BA, Amsterdam, The Netherlands

3: Laboratory of Neuropathology, Department of Imaging and Pathology, and Leuven Brain Institute, KU Leuven, 3000 Leuven, Belgium

4: Department of Neurology, University Hospitals Leuven, 3000 Leuven, Belgium and Laboratory of Neurobiology, Department of Neuroscience, KU Leuven, 3000 Leuven, Belgium

5: Department of Neurology, Ulm University, Ulm, 89081, Germany.

6: Department of Geriatrics, University Medical Center Göttingen, Göttingen, Germany

7: Department of Neurology, University of Halle, Halle, 06120, Germany

8: Department of Pathology, UZ Leuven, 3000 Leuven, Belgium

Correspondence to: Sanne Vermorgen

Department of Pathology, Amsterdam UMC, De Boelelaan 1117,1081 HV Amsterdam, The Netherlands

s.m.vermorgen@amsterdamumc.nl

Supplementary Methods:

Additional details of neuropathological tissue preparation:

1. KUL cohort:

At fresh autopsy, the brains were weighed and inspected for atherosclerosis, infarctions, bleedings and atrophy. One hemisphere was sliced and frozen while the other hemisphere was left intact for formalin fixation (4% formalin) during 4 to 6 weeks. After fixation, samples from the anterior MTL, posterior MTL, middle frontal gyrus, occipital cortex, midbrain, pons, medulla oblongata, cerebellum and basal ganglia were collected. These samples were embedded in paraffin and sectioned into 5 µm slices for hematoxylin-eosin staining (HE) and immunohistochemical staining. Epitope retrieval was done with citrate buffer (pH 6; Envision Flex Target Retrieval Solution, Dako, K8005) at 97 degrees Celsius for 10 minutes. For Aβ and α-synuclein staining, 5 minutes incubation in 98% to 100% formic acid was done at room temperature. The slides were incubated with the primary antibody overnight in a humid chamber at room temperature, and the next day incubation with the secondary antibodies was done, either using anti-mouse HRP-linked secondary antibodies diluted in Tris-HCl-based diluent for 30 minutes, or with the VECTASTAIN ABC-HRP kit (Vector Laboratories, involving a 30 minutes incubation with the biotinylated secondary antibody followed by a 30 minute incubation with the ABC reagent at room temperature. DAB solution, applied for 10 minutes, was used to visualize the binding (Liquid DAB+ Substrate Chromogen System, DAKO, catalog no. K3468). Hematoxylin was used for counterstaining, using an autostainer. Coverslips were mounted automatically using a cover slipper (Leica Microsystems). Each case was assessed and diagnosed by a qualified neuropathologist, for phosphorylated Tau (tangles , neuropil threads, Argyrophilic grains and astrocytic Tau), Amyloid Beta (plaques and cerebral amyloid angiopathy), Neuritic plaques, phosphorylated TDP43 (threads, neuronal cytoplasmic inclusions, neuronal intraneuronal inclusions), alphasynuclein deposits (Lewy bodies, Papp-Lantos bodies), presence of infarctions or bleedings, presence of malignancies. Established protocols were used for the assessment of Aβ plaque deposition, Braak stages for neurofibrillary tangles and Consortium to Establish a Registry for Alzheimer's Disease (CERAD) score for neuritic plaques.[1-3] The neuropathological diagnosis of Alzheimer's Disease Neuropathological Changes (ADNC) was performed using the published guidelines with the ABC score as a measure for ADNC severity.[4] ARTAG and AGD were assessed using the AT8 staining.[5-6] LATE-NC and FTLD-TDP were assessed using the phosphorylated TDP-43 antibody according to published guidelines.[7-8] α-synuclein pathology was assessed using the 5G4 anti aggregates α-synuclein antibody according to published guidelines.[9]

2. NBB cohort

At fresh autopsy, the brain was weighed, photographed and assessed for infarcts, atrophy, symmetry and atherosclerosis. The left hemisphere was sampled for regions of interest (frozen or formalin fixed after collection) according to the protocol determined by the brain donation registration. The right hemisphere was left intact and formalin fixed for 4-5 weeks (4% formalin). After fixation, the right hemisphere was sectioned in 1 cm thick sections and evaluated for infarcts, lesions, atrophy and signs of neurodegenerative disease. Samples were collected from at least the middle frontal gyrus, middle temporal gyrus, anterior cingulate cortex, parietal cortex, motor cortex, occipital cortex, basal ganglia, anterior hippocampus, middle hippocampus, amygdala, thalamus, midbrain, pons, medulla oblongata, cerebellum and meninges. These samples were embedded in paraffin and sliced in 4-6µm sections. HE stain and Gallyas silver stain was done, and for immunohistochemistry endogenous peroxidase activity was blocked in 0.3% H_2O_2 in phosphate buffered saline at a pH of 7.0 for 30 minutes, except for α-synuclein staining. A heated sodium-citrate buffer (10mM/L, pH 6.0) was used for antigen retrieval. Primary antibody incubation was done at 4 degrees Celsius overnight. The next day, EnVision detection system (goat anti-mouse/rabbit horseradish peroxidase (HRP), DAKO, Heverlee, Belgium) was used for 40 minutes incubation at room temperature. PBS was used to rinse the sections between incubation steps. Five minutes incubation of the sections was done with chromogen 3,3'-DAB (DAB, EnVision Detection system/HRP, DAKO, Heverlee, Belgium) to visualize the binding. Hematoxylin was used for counterstaining. Slides were dehydrated and mounted using the non-aqueous mounting medium Quick-D

(Klinipath, Duiven, Netherlands). Slides were evaluated by an qualified neuropathologist, using internationally accepted guidelines.[1-9]

1. Braak H, Braak E. Neuropathological stageing of Alzheimer-related changes. Acta Neuropathol. 1991; 82(4): 239-259. doi:10.1007/BF00308809
2. Thal DR, Rüb U, Orantes M, Braak H. Phases of Aß -deposition in the human brain and its relevance for the development of AD. Neurology. 2002; 58(12): 1791-1800. doi:10.1212/wnl.58.12.1791
3. Mirra SS, Heyman A, McKeel D, et al. The Consortium to Establish a Registry for Alzheimer's Disease (CERAD). Part II. Standardization of the neuropathologic assessment of Alzheimer's disease. Neurology. 1991; 41(4): 479-486. doi:10.1212/wnl.41.4.479
4. Montine TJ, Phelps CH, Beach TG, et al. National Institute on Aging-Alzheimer's Association guidelines for the neuropathologic assessment of Alzheimer's disease: a practical approach. Acta Neuropathol. 2012; 123(1): 1-11. doi:10.1007/s00401-011-0910-3
5. Kovacs GG, Ferrer I, Grinberg LT, et al. Aging-related tau astrogliopathy (ARTAG): harmonized evaluation strategy. Acta Neuropathol. 2016; 131(1): 87-102. doi:10.1007/s00401-015-1509-x
6. Braak H, Braak E. Argyrophilic grain disease: frequency of occurrence in different age categories and neuropathological diagnostic criteria. J Neural Transm (Vienna). 1998; 105(8-9): 801-819. doi:10.1007/s007020050096
7. Nelson PT, Lee EB, Cykowski MD, et al. LATE-NC staging in routine neuropathologic diagnosis: an update. Acta Neuropathol. 2023; 145(2): 159-173. doi:10.1007/s00401-022-02524-2
8. Mackenzie IR, Neumann M, Barborie A, et al. A harmonized classification system for FTLD-TDP pathology. Acta Neuropathol. 2011;122:111–113. doi: 10.1007/s00401-011-0845-8.
9. Braak H, Del Tredici K, Rub U, de Vos RA, Jansen Steur EN, Braak E. Staging of brain pathology related to sporadic Parkinson's disease. *Neurobiol Aging*. Mar-Apr 2003;24(2):197-211. doi:10.1016/s0197-4580(02)00065-9

Supplementary tables and figures:

Supplementary table 1a: Descriptive statistics for the seven diagnostic categories for the young cohort (48-65 years of age)

Diagnostic Category	% Male	Mean Age	Mean Brain Weight	Median CDR	Median Braak-LBD (Mode)	% α-Synuclein
CONTROL	38.9%	58.39 (5.10)	1221.35 (157.13)	0	0 (0)	0.0%
OTHER ND-	25%	58.05 (4.33)	1204.32 (208.40)	0	0 (0)	0.0%
AD-NC	57.1%	58.82 (4.82)	1296.78 (154.66)	0	0 (0)	0.0%
ALZHEIMER'S DISEASE	63.6%	60.82 (3.19)	1178.55 (186.14)	3	0 (0)	61.9%
α-SYNUCLEINOPATHY	66.7%	59.73 (5.05)	1348.93 (110.46)	0.5	5 (6)	100%
TDP PROTEINOPATHY	63.6%	60.58 (4.37)	1200.82 (223.83)	1.5	0 (0)	6.1%
TAUOPATHY	66.7%	61.67 (2.99)	1268.42 (174.09)	2	0 (0)	16.7%
OTHER ND+	83.3%	56.33 (3.57)	1191.56 (125.12)	0	0 (0)	11.1%

Diagnostic Category	% TDP43	% LATE	% ALS	% FTLD-TDP43	% ALS-FTLD-TDP43
CONTROL	0.0%	0.0%	0.0%	0.0%	0.0%
OTHER ND-	0.0%	0.0%	0.0%	0.0%	0.0%
AD-NC	0.0%	0.0%	0.0%	0.0%	0.0%
ALZHEIMER'S DISEASE	36.4%	36.4%	0.0%	0.0%	0.0%
α -SYNUCLEINOPATHY	0.0%	0.0%	0.0%	0.0%	0.0%
TDP PROTEINOPATHY	100%	6.1%	36.4%	45.5%	12.1%
TAUOPATHY	25.0%	16.7%	0.0%	8.3%	0.0%
OTHER ND+	0.0%	0.0%	33.3%	0.0%	0.0%

Diagnostic Category	% multiproteinopathy	% (micro)infarcts	% malignancy
CONTROL	0%	22.2%	5.6%
OTHER ND-	0%	25%	20%
AD-NC	0%	21.4%	3.6%
ALZHEIMER'S DISEASE	81.8%	18.2%	4.8%
α -SYNUCLEINOPATHY	100%	26.7%	6.7%
TDP PROTEINOPATHY	100%	6.3%	0.0%
TAUOPATHY	41.7%	16.7%	0.0%
OTHER ND+	22.2%	22.2%	0.0%

Diagnostic Category	Median Aβ phase (Mode)	Median Braak NFT (Mode)	Median A-score (Mode)	Median B-score (Mode)	Median C-score (Mode)
CONTROL	0 (0)	1 (1)	0 (0)	1 (1)	0 (0)
OTHER ND-	0 (0)	1 (1)	0 (0)	1 (1)	0 (0)
AD-NC	1 (1)	1 (1)	1 (1)	1 (1)	0 (0)
ALZHEIMER'S DISEASE	5 (5)	6 (6)	3 (3)	3 (3)	3 (3)
α -SYNUCLEINOPATHY	0 (0)	1 (1)	0 (0)	1 (1)	0 (0)
TDP PROTEINOPATHY	0 (0)	1 (1)	0 (0)	1 (1)	0 (0)
TAUOPATHY	1 (1)	1 (1)	1 (1)	1 (1)	0 (0)
OTHER ND+	0 (0)	1 (1)	0 (0)	1 (1)	0 (0)

Diagnostic Category	% CAA	Median CAA stage (Mode)	% CAA type 1	% CAA type 2
CONTROL	5.6%	0 (0)	0.0%	5.6%
OTHER ND-	20%	0 (0)	10%	10%
AD-NC	35.7%	0 (0)	21.4%	14.3%
ALZHEIMER'S DISEASE	100%	1 (1)	40.9%	59.1%
α -SYNUCLEINOPATHY	35.7%	0 (0)	7.1%	28.6%
TDP PROTEINOPATHY	9.1%	0 (0)	3%	6.1%
TAUOPATHY	50.0%	0.5 (0)	8.3%	41.7%
OTHER ND+	22.2%	0 (0)	0.0%	22.2%

Supplementary table 1b: Descriptive statistics for the seven diagnostic categories for the NBB 66+ cohort

Diagnostic Category	N	% Male	Mean Age	Mean Brain Weight	Median Braak-LBD (Mode)	% α-Synuclein
CONTROL	14	71.4%	78.79 (10.50)	1286.5 (152.47)	0 (0)	0.0%
OTHER ND-	5	40.0%	78.2 (8.88)	1099.6 (102.78)	0 (0)	0.0%
AD-NC	58	48.3%	81.14 (8.11)	1176.03 (161.46)	0 (0)	0.0%
ALZHEIMER'S DISEASE	108	50.9%	83.01 (9.35)	1163.97 (135.59)	0 (0)	6.5%
α-SYNUCLEINOPATHY	153	58.8%	80.31 (8.07)	1228.8 (205.78)	6 (6)	4.5%
TDP PROTEINOPATHY	22	54.5%	78.05 (9.529)	1114.05 (165.64)	0 (0)	3.6%
TAUOPATHY	28	50.0%	77.07 (5.33)	1175.96 (142.64)	0 (0)	3.6%
OTHER ND+	9	55.6%	78.89 (6.01)	1154.78 (93.55)	0 (0)	0.0%

Diagnostic Category	% TDP43	% LATE	% ALS	% FTLD-TDP43	% ALS-FTLD-TDP43
CONTROL	0.0%	0.0%	0.0%	0.0%	0.0%
OTHER ND-	0.0%	0.0%	0.0%	0.0%	0.0%
AD-NC	0.0%	0.0%	0.0%	0.0%	0.0%
ALZHEIMER'S DISEASE	23.1%	23.1%	0.0%	0.0%	0.0%
α -SYNUCLEINOPATHY	16.3%	16.3%	0.0%	0.0%	0.0%
TDP PROTEINOPATHY	100.0%	9.1%	9.1%	63.6%	9.1%
TAUOPATHY	7.1%	3.6%	0.0%	3.6%	0.0%
OTHER ND+	0.0%	0.0%	0.0%	0.0%	0.0%

Diagnostic Category	% (micro)infarcts	% malignancy
CONTROL	28.6%	0.0%
OTHER ND-	40.0%	20.0%
AD-NC	13.8%	3.4%
ALZHEIMER'S DISEASE	8.3%	3.7%
α -SYNUCLEINOPATHY	11.1%	5.2%
TDP PROTEINOPATHY	4.5%	0.0%
TAUOPATHY	3.6%	0.0%
OTHER ND+	11.1%	0.0%

Diagnostic Category	Median Aβ phase (Mode)	Median Braak NFT (Mode)	Median A-score (Mode)	Median B-score (Mode)	Median C-score (Mode)
CONTROL	0 (0)	1 (1)	0 (0)	1 (1)	0 (0)
OTHER ND-	0 (0)	1 (1)	0 (0)	1 (1)	0 (0)
AD-NC	0 (0)	2 (2)	1 (1)	1 (1)	0 (0)
ALZHEIMER'S DISEASE	5 (5)	5 (6)	3 (3)	3 (3)	2 (3)
α -SYNUCLEINOPATHY	3 (1)	3 (3)	2 (1)	2 (1)	0 (0)
TDP PROTEINOPATHY	3 (3)	2 (2)	1 (1)	1 (1)	0 (0)
TAUOPATHY	1 (3)	2 (3)	1 (1)	1 (1)	0 (0)
OTHER ND+	0 (0)	2 (2)	1 (1)	1 (1)	0 (0)

Diagnostic Category	% CAA	Median CAA stage (Mode)	% CAA type 1	% CAA type 2
CONTROL	0.0%	0 (0)	0.0%	0.0%
OTHER ND-	20.0%	0 (0)	0.0%	20.0%
AD-NC	31.1%	1 (1)	19.0%	12.1%
ALZHEIMER'S DISEASE	73.2%	1 (1)	46.3%	26.9%
α -SYNUCLEINOPATHY	39.9%	1 (1)	21.6%	18.3%
TDP PROTEINOPATHY	45.4%	1 (1)	22.7%	22.7%
TAUOPATHY	50.0%	1 (1)	14.3%	35.7%
OTHER ND+	22.2%	2 (2)	0.0%	22.2%

Supplementary table 1c: Descriptive statistics for the seven diagnostic categories for KUL the 66+ cohort

Diagnostic Category	N	% Male	Mean Age	Mean Brain Weight	Median Braak-LBD (Mode)	% α-Synuclein
CONTROL	14	71.4%	70.14 (4.26)	1313.08 (133.79)	0 (0)	0.0%
OTHER ND-	15	60.0%	74.13 (6.65)	1214.2 (129.94)	0 (0)	0.0%
AD-NC	60	55.0%	74.62 (7.23)	1240.5 (160.96)	0 (0)	0.0%
ALZHEIMER'S DISEASE	58	43.1%	80.03 (8.08)	1141.88 (172.50)	0 (0)	0.0%
α-SYNUCLEINOPATHY	58	67.2%	78.79 (7.16)	1230.86 (194.56)	5 (6)	100.0%
TDP PROTEINOPATHY	44	52.3%	77.25 (8.00)	1144.29 (204.86)	0 (0)	22.7%
TAUOPATHY	18	61.1%	76.78 (7.55)	1191.46 (85.81)	0 (0)	5.6%
OTHER ND+	1	100.0%	73	1366	0 (0)	0.0%

Diagnostic Category	% TDP43	% LATE	% ALS	% FTLD-TDP43	% ALS-FTLD-TDP43
CONTROL	0.0%	0.0%	0.0%	0.0%	0.0%
OTHER ND-	0.0%	0.0%	0.0%	0.0%	0.0%
AD-NC	0.0%	0.0%	0.0%	0.0%	0.0%
ALZHEIMER'S DISEASE	46.6%	36.2%	0.0%	1.7%	0.0%
α -SYNUCLEINOPATHY	22.4%	19.0%	0.0%	0.0%	0.0%
TDP PROTEINOPATHY	97.7%	18.2%	36.4%	31.8%	6.8%
TAUOPATHY	11.1%	11.1%	0.0%	0.0%	0.0%
OTHER ND+	0.0%	0.0%	0.0%	0.0%	0.0%

Diagnostic Category	% (micro)infarcts	% malignancy
CONTROL	14.3%	0.0%
OTHER ND-	73.7%	13.3%
AD-NC	28.3%	8.3%
ALZHEIMER'S DISEASE	24.1%	1.7%
α -SYNUCLEINOPATHY	22.4%	0.0%
TDP PROTEINOPATHY	11.4%	4.5%
TAUOPATHY	11.1%	0.0%
OTHER ND+	0.0%	0.0%

Diagnostic Category	Median Aβ phase (Mode)	Median Braak NFT (Mode)	Median A-score (Mode)	Median B-score (Mode)	Median C-score (Mode)
CONTROL	0 (0)	1 (1)	0 (0)	1 (1)	0 (0)
OTHER ND-	0 (0)	1 (1)	0 (0)	1 (1)	0 (0)
AD-NC	2 (2)	2 (1)	1 (1)	1 (1)	0 (0)
ALZHEIMER'S DISEASE	5 (5)	4 (4)	3 (3)	3 (3)	2 (2)
α -SYNUCLEINOPATHY	4 (5)	3 (2)	3 (3)	2 (1)	0 (0)
TDP PROTEINOPATHY	1 (0)	2 (1)	1 (1)	1 (1)	0(0)
TAUOPATHY	0.5 (0)	2 (2)	0.5 (0)	1 (1)	0(0)
OTHER ND+	0 (0)	2 (2)	0 (0)	1 (1)	0 (0)

Diagnostic Category	% caa	Median CAA stage (Mode)	% CAA type 1	% CAA type 2
CONTROL	0.0%	0 (0)	0.0%	0.0%
OTHER ND-	6.7%	0 (0)	0.0%	6.7%
AD-NC	53.3%	0.5 (0)	10.0%	43.3%
ALZHEIMER'S DISEASE	91.4%	2 (2)	65.5%	25.9%
α -SYNUCLEINOPATHY	74.2%	1 (2)	34.5%	39.7%
TDP PROTEINOPATHY	38.7%	0 (0)	11.4%	27.3%
TAUOPATHY	33.3%	0 (0)	0.0%	33.3%
OTHER ND+	100%	1 (1)	0.0%	100%

Supplementary table 2: differences between KUL and NBB datasets (48-65 cohort)

Variable	Mann-Whitney	P-value	Direction of difference
AGE	1924.5	< 0.001	KUL YOUNGER
CDR	867	< 0.001	NBB higher CDR
Aβ PHASE	2303.5	0.004	NBB HIGHER β-AMYLOÏD PLAQUE PHASE
CAA STAGE	2316	0.008	NBB higher CAA stage
A-SCORE	2243	0.002	NBB HIGHER A-SCORE
C-SCORE	2371.5	<0.001	NBB higher C-score

Variable	Chi square	P-value	Direction of difference
TDP-43	11.36	<0.001	KUL MORE TDP-43
CAA	6.54	0.011	NBB more CAA
α-SYNUCLEIN	29.32	<0.001	NBB MORE α-SYNUCLEIN
NEURODEGENERATIVE DISEASE	4.485	0.034	NBB more neurodegenerative disease

Comparison KUL and NBB dataset. Only significant differences are displayed. Abreviations: age: age at death; CDR: clinical dementia rating; Ab plaques: Amyloid beta plaque phase (Thal, 2002); McKeith: DLB stage (McKeith, 2005); CAA stage: cerebral amyloid angiopathy stage (Thal, 2008); A score: amyloid plaque grading ABC score (Montine, 2012); C-score: neuritic plaque severity (Montine, 2012); TDP-43: presence of TDP-43 pathology, regardless of type; TDP-ALS: presence of ALS-type TDP43 pathology; CAA: presence of cerebral amyloid angiopathy regardless of type and stage; alphasyn: presence of alphasynuclein pathology, regardless of type and grading.

Supplementary table 3: list of antibodies

Aβ	Anti $A\beta_{17-24,}$ 4G8, Signet Dedham, MA, USA; 1:5000, formic acid pretreatment
ptau	Anti p-$tau^{Ser202/Thr205}$ mouse antibody AT8, Pierce Biotechnology, Rockford, IL, USA 1:1000; pretreatment
α-synuclein	- LB509 mouse antibody, Zymed, Thermo Fisher Scientific, Bleiswijk, The Netherlands (for NBB cases)<br>- Anti aggregates αSyn, 5G4, Merck-Millipore (for KUL cases)
pTDP43	Anti $pTDP43^{Ser409/Ser410}$, mouse antibody, Cosmo Bio, Tokyo, Japan

Supplementary table 4: results of univariate analysis (including non-significant results)

a) 48-65 cohort: variables tested for association with ARTAG, lobar ARTAG (only for ARTAG cases) and with ARTAG severity (only for ARTAG cases):

	ARTAG		ARTAG severity		Lobar ARTAG	
Age	*U* = 2208.5	*P* = 0.017	*KW H* = 2.841	*P* = 0.242	*U* = 63.5	*P* = 0.866
Neurodegenerative disease	χ^2 = 7.039	*P* = 0.008	/	/	χ^2 = 0.383	*P* = 0.536
Sex	χ^2 = 0.102	*P* = 0.749	/	/	χ^2 = 1.192	*P* = 0.275
Brain weight	*U* = 1658	*P* = 0.977	*KW H* = 3.534	*P* = 0.171	*U* = 56	*P* = 0.572
Presence of (micro)infarcts	χ^2 = 0.212	*P* = 0.645	/	/	χ^2 = 1.742	*P* = 0.187
Multiproteinopathy	χ^2 = 2.834	*P* = 0.092	*KW H* = 2.304	*P* = 0.316	χ^2 = 2.365	*P* = 0.124
Braak-NFT stage	*U* = 1933	*P* = 0.236	*KW H* =1.480	*P* = 0.477	*U* = 96	*P* = 0.094
Aβ phase	*U* = 1767.5	*P* = 0.746	*KW H* = 2.303	*P* = 0.316	*U* = 69	*P* = 0.910
A-score	*U* = 1756.5	*P* = 0.787	*KW H* = 2.339	*P* = 0.310	*U* = 67.5	*P* = 0.955
B-score	*U* = 1954	*P* = 0.155	*KW H* = 3.193	*P* = 0.203	*U* = 101.5	*P* = 0.041
C-score	*U* = 1849.5	*P* = 0.306	*KW H* = 0.793	*P* = 0.673	*U* = 83	*P* = 0.364
CAA	χ^2 = 0.966	*P* = 0.326	/	/	χ^2 = 0.001	*P* = 0.973
CAA-stage	*U* = 1849.5	*P* = 0.244	*KW H* = 1.825	*P* = 0.401	*U* = 76	*P* = 0.611
α-synuclein	χ^2 = 1.729	*P* = 0.188	/	/	χ^2 = 3.128	*P* = 0.077
TDP-43	χ^2 = 1.682	*P* = 0.195	/	/	χ^2 = 0.078	*P* = 0.780

U: Independent samples Mann-Whitney U test, x^2: Pearson Chi-square test, *KW H*: independent samples Kruskal-Wallis test

b) 66+ cohort: variables tested for association with ARTAG:

	KUL		NBB	
	ARTAG		ARTAG	
Age	U = 8570	P < 0.001	U = 17454.5	P < 0.001
Neurodegenerative disease	χ^2 = 2.056	P = 0.152	χ^2 = 0.021	P = 0.884
Sex	χ^2 = 0.269	P = 0.604	χ^2 = 1.717	P = 0.190
Brain weight	U = 3977.5	P = 0.504	U = 14021.5	P = 741
Presence of (micro)infarcts	χ^2 = 0170	P = 0.680	χ^2 = 0.277	P = 0.598
Braak-NFT stage	U = 6795	P = 0.809	U = 12720	P = 0.436
Aβ phase	U = 6342	P = 0.545	U = 7333	P = 0.945
A-score	U = 6347.5	P = 0.541	U = 13767	P = 0.390
B-score	U = 6387	P = 0.567	U = 13843.5	P = 0.173
C-score	U = 6407.5	P = 0.587	U = 13048.5	P = 0.960
CAA	χ^2 = 0.011	P = 0.918	χ^2 = 0.965	P = 0.326
CAA-stage	U = 6136	P = 0.731	U = 1289	P = 0.703
α-synuclein	χ^2 = 0.000	P = 0.998	χ^2 = 1.758	P = 0.185
TDP-43	χ^2 = 0.008	P = 0.930	χ^2 = 2.749	P = 0.097

U: Independent samples Mann-Whitney U test, x^2: Pearson Chi-square test, $KW\ H$: independent samples Kruskal-Wallis test

Supplementary figure 1: distribution ordinal variables for each diagnostic group (48-65 cohort only)

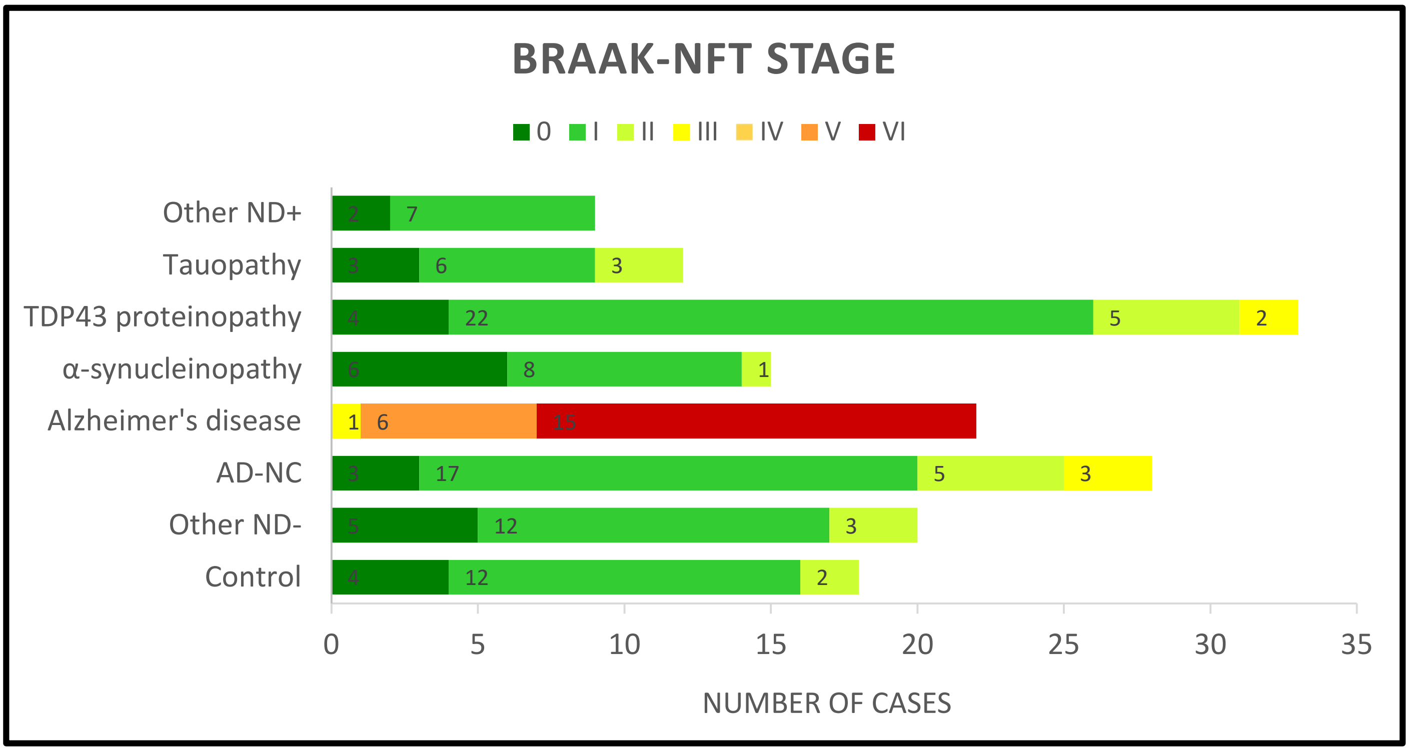

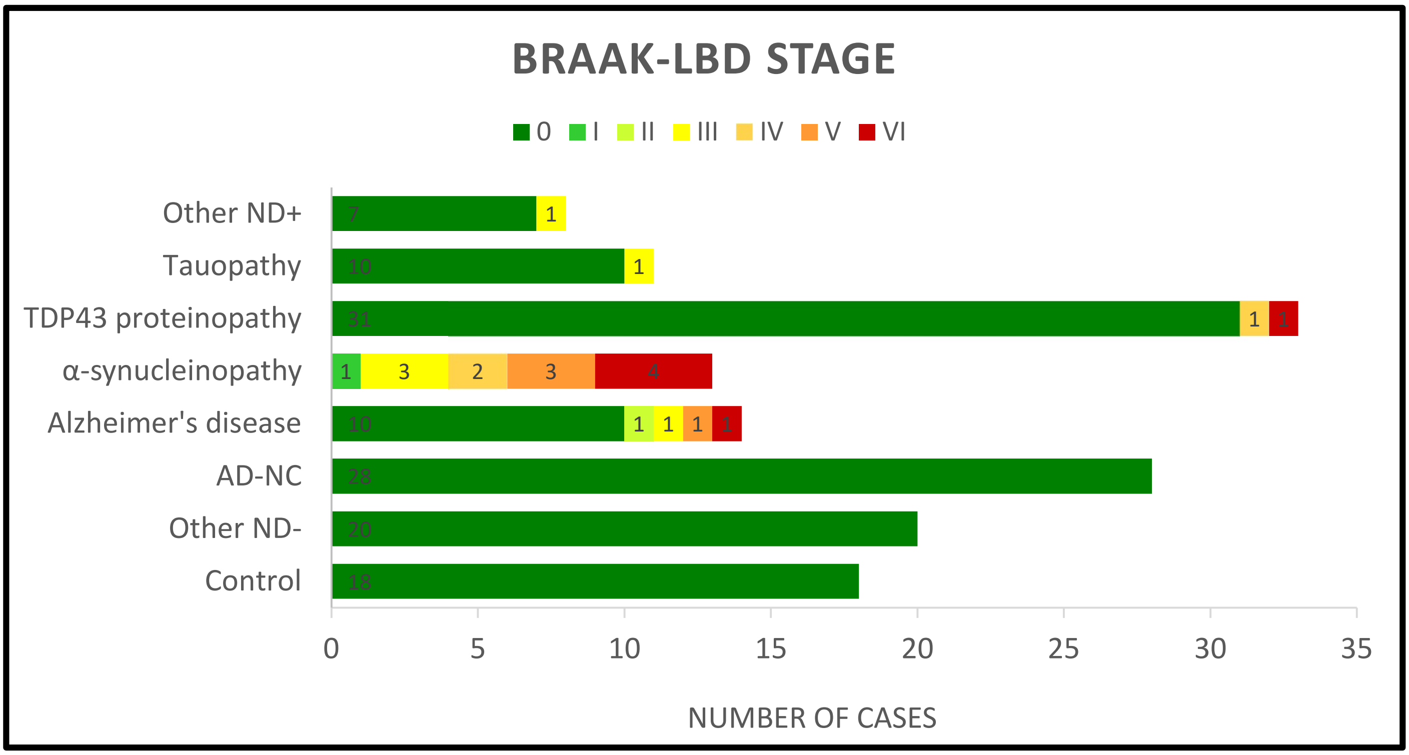

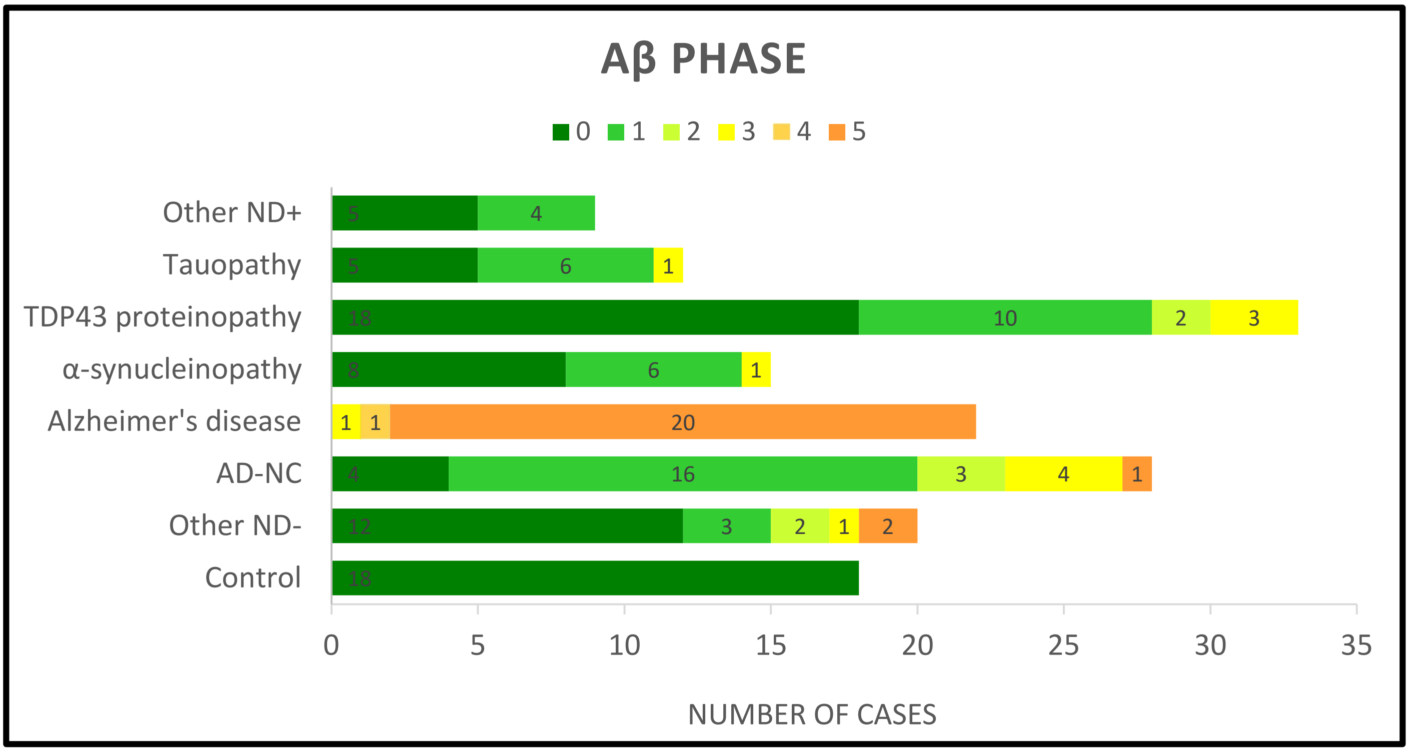
Aβ PHASE
0
1
2
3
4
5
Other ND+
Tauopathy
TDP43 proteinopathy
α-synucleinopathy
Alzheimer's disease
AD-NC
Other ND-
Control
5
4
5
6
1
18
10
2
3
8
6
1
1
1
20
4
16
3
4
1
12
3
2
1
2
18
0
5
10
15
20
25
30
35
NUMBER OF CASES

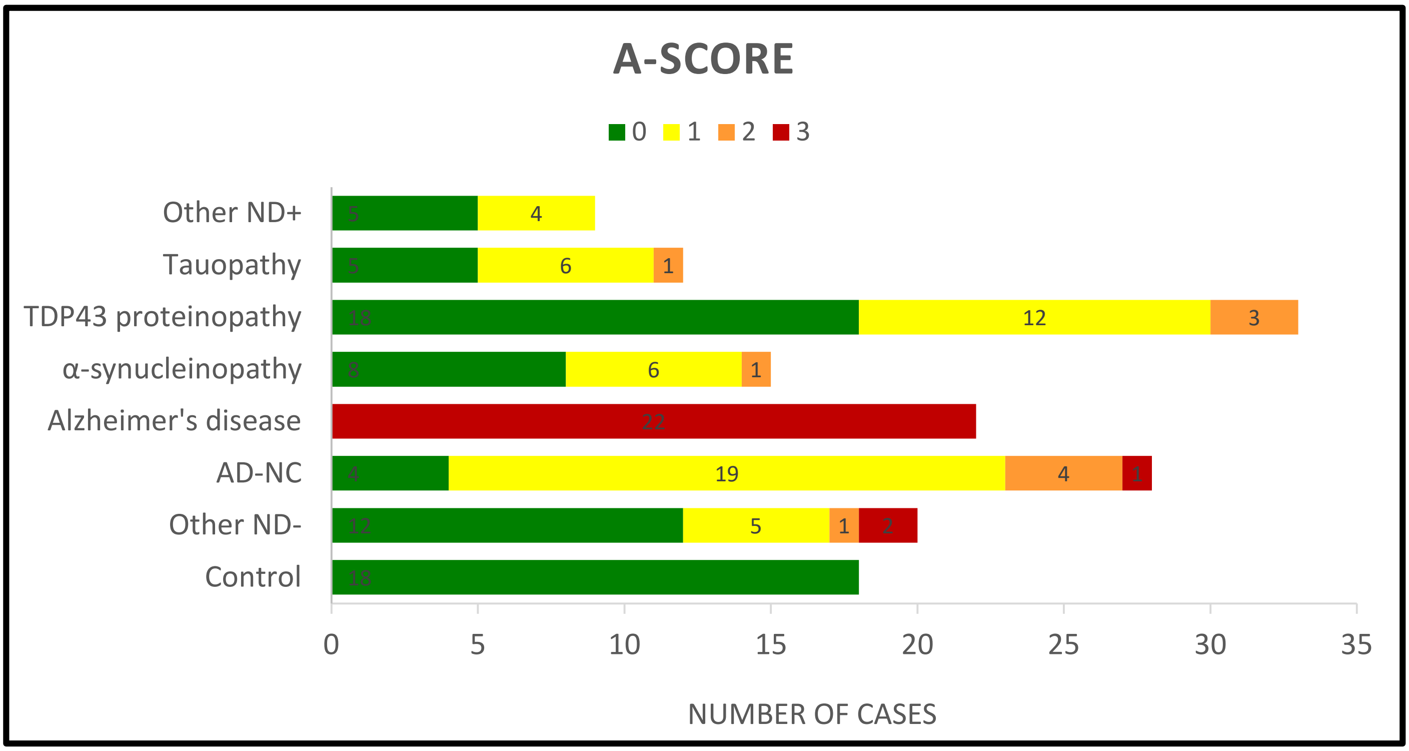
A-SCORE
0
1
2
3
Other ND+
Tauopathy
TDP43 proteinopathy
α-synucleinopathy
Alzheimer's disease
AD-NC
Other ND-
Control
5
4
5
6
1
18
12
3
8
6
1
22
4
19
4
1
12
5
1
2
18
0
5
10
15
20
25
30
35
NUMBER OF CASES

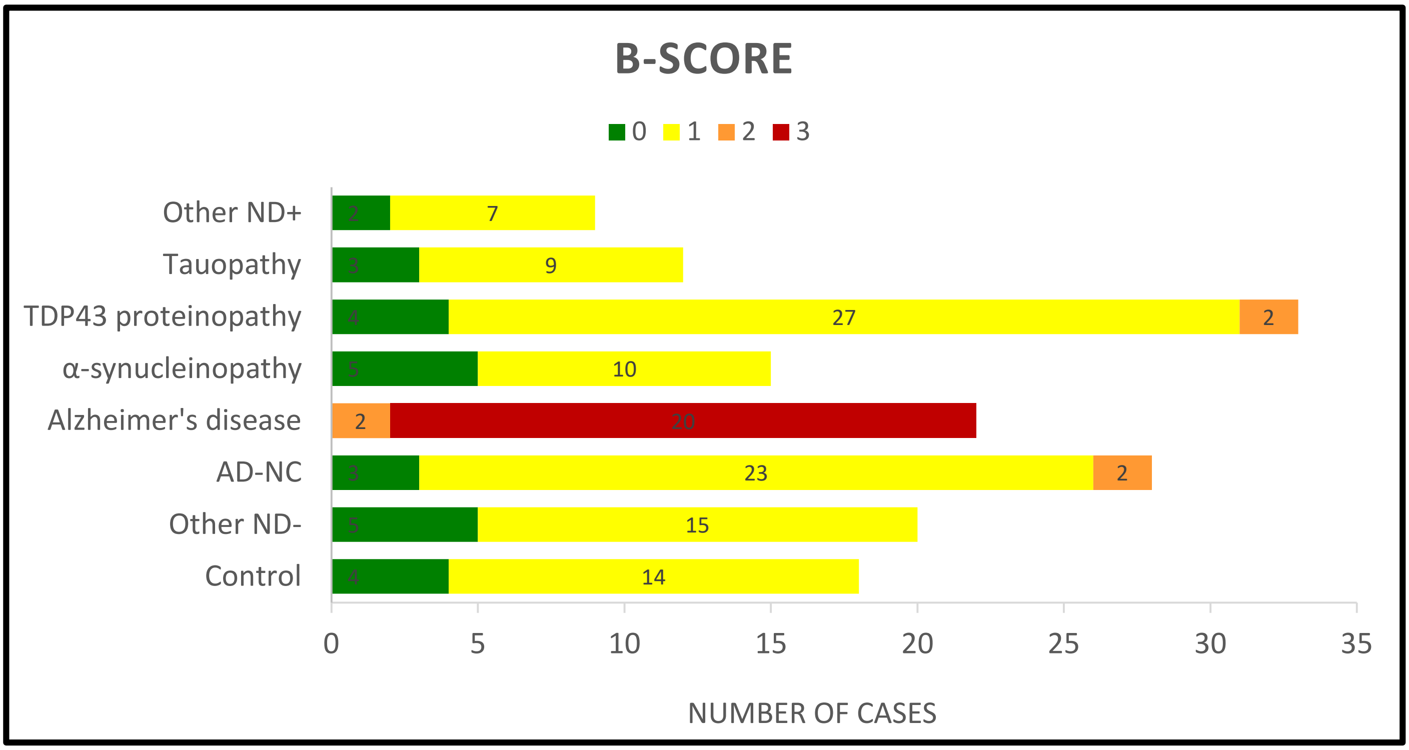
B-SCORE
0
1
2
3
Other ND+
Tauopathy
TDP43 proteinopathy
α-synucleinopathy
Alzheimer's disease
AD-NC
Other ND-
Control
2
7
3
9
4
27
2
5
10
2
20
3
23
2
5
15
4
14
0
5
10
15
20
25
30
35
NUMBER OF CASES

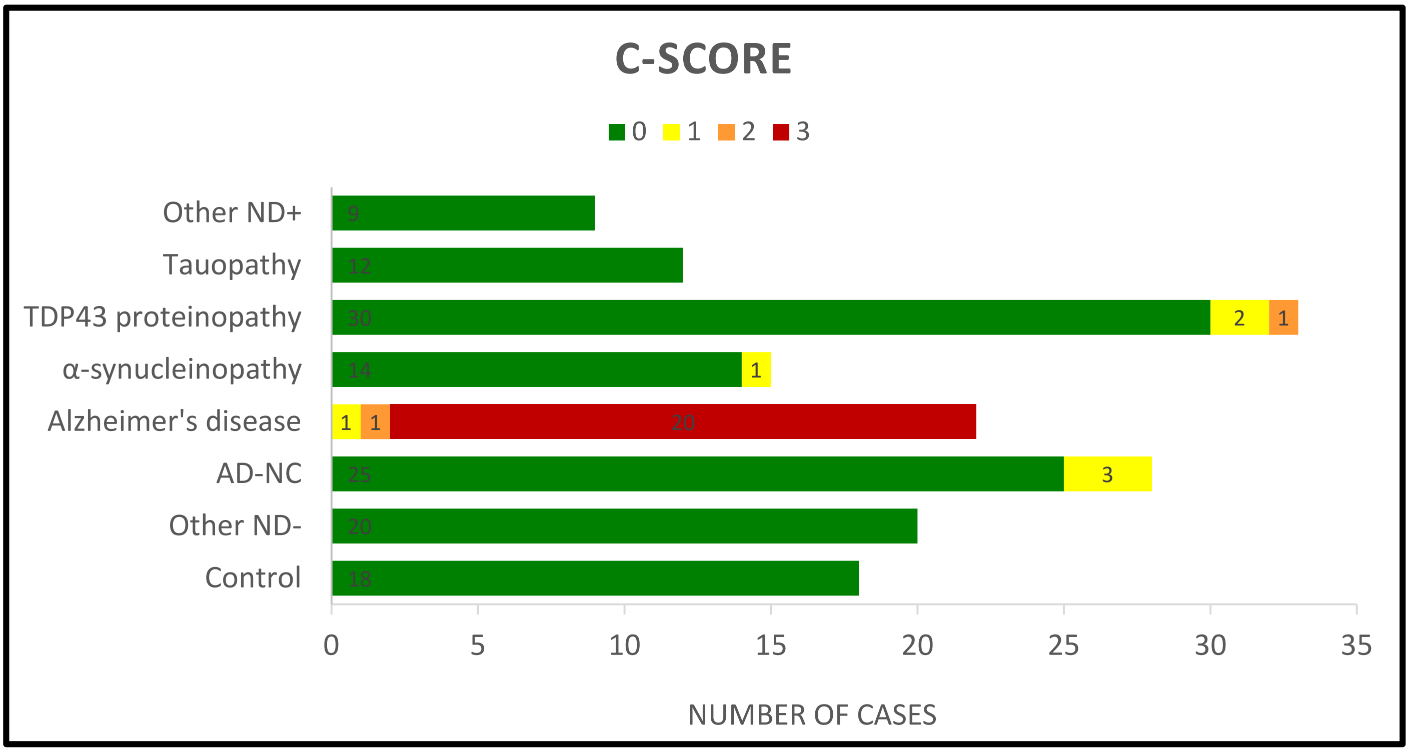
C-SCORE
0
1
2
3
Other ND+
Tauopathy
TDP43 proteinopathy
α-synucleinopathy
Alzheimer's disease
AD-NC
Other ND-
Control
9
12
30
2
1
14
1
1
1
20
25
3
20
18
0
5
10
15
20
25
30
35
NUMBER OF CASES

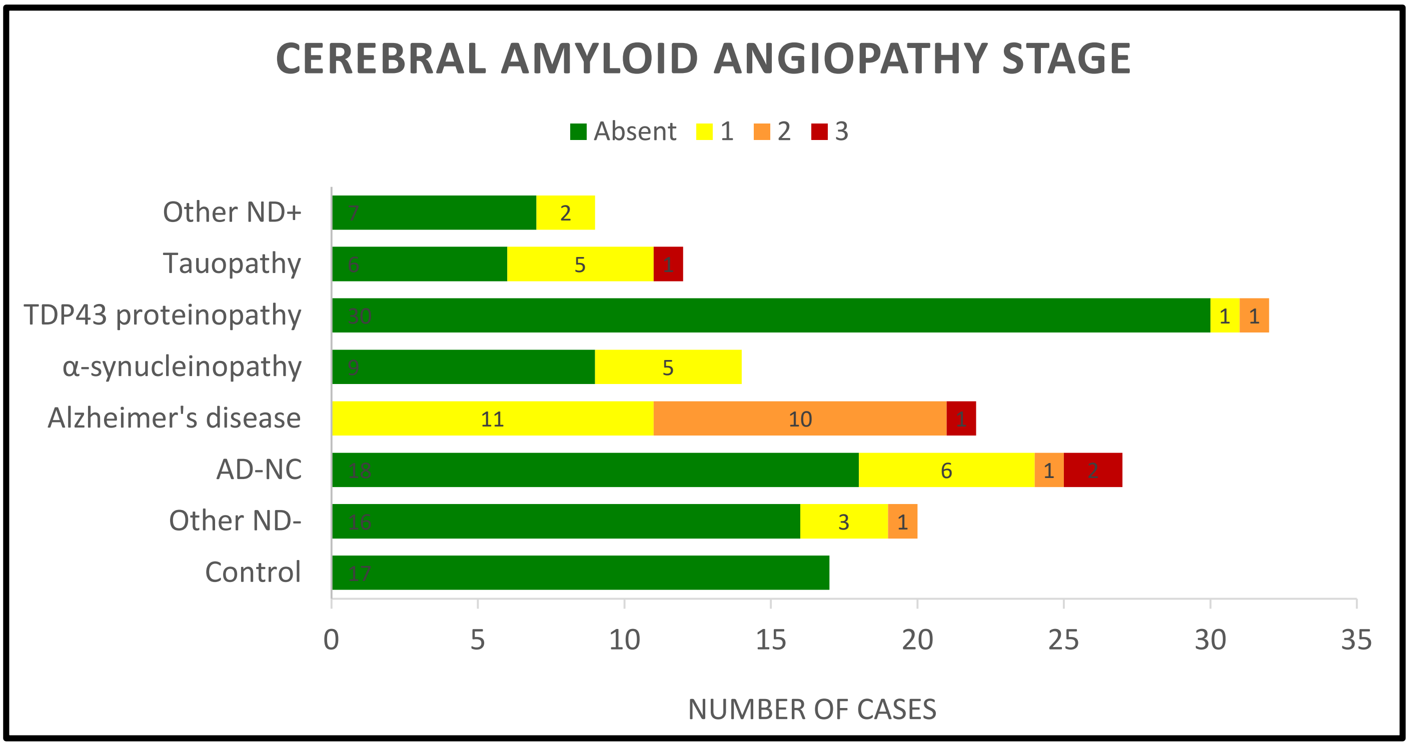
CEREBRAL AMYLOID ANGIOPATHY STAGE
Absent
1
2
3
Other ND+
Tauopathy
TDP43 proteinopathy
α-synucleinopathy
Alzheimer's disease
AD-NC
Other ND-
Control
7
2
6
5
1
30
1
1
9
5
11
10
1
18
6
1
2
16
3
1
17
0
5
10
15
20
25
30
35
NUMBER OF CASES

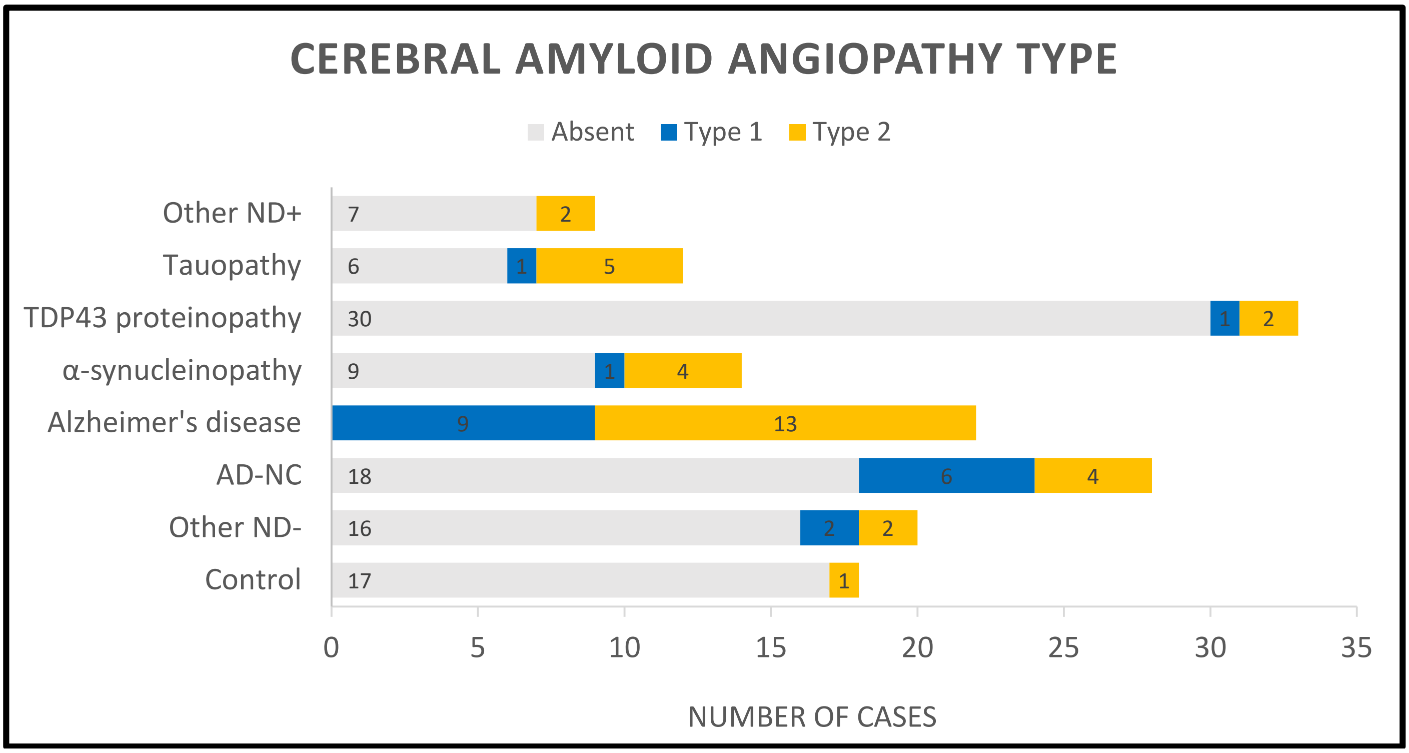
CEREBRAL AMYLOID ANGIOPATHY TYPE
Absent
Type 1
Type 2
Other ND+
Tauopathy
TDP43 proteinopathy
α-synucleinopathy
Alzheimer's disease
AD-NC
Other ND-
Control
7
2
6
1
5
30
1
2
9
1
4
9
13
18
6
4
16
2
2
17
1
0
5
10
15
20
25
30
35
NUMBER OF CASES

Supplementary figure 2: ARTAG according to subcategories pTDP proteinopathy, with tentative analysis results (48-65 cohort)

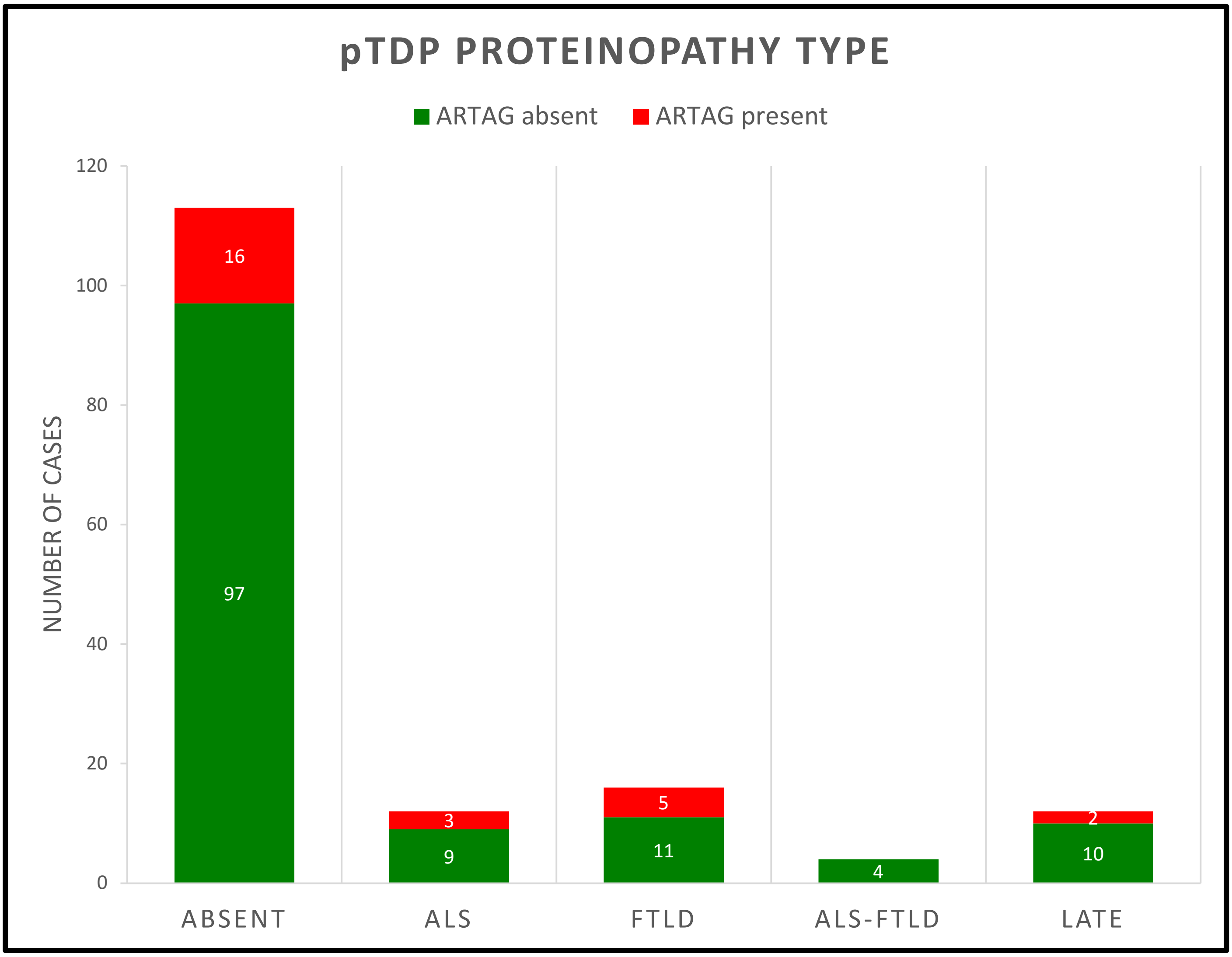

ARTAG distribution in TDP subtypes (48-65 cohort)

		ARTAG					
		absent		present		Total	
		N	%	N	%	N	%
TDP subtypes	ALS	9_a	26,5%	3_a	30,0%	12	27,3%
	FTLD	11_a	32,4%	5_a	50,0%	16	36,4%
	ALS-FTLD	4_a	11,8%	0_a	0,0%	4	9,1%
	LATE	10_a	29,4%	2_a	20,0%	12	27,3%
Total		34	100,0%	10	100,0%	44	100,0%

Each subscript letter denotes a subset of ARTAG categories whose column proportions do not differ significantly from each other at the ,05 level.

Supplementary figure 3: ARTAG according to LATE-NC stages (48-65 cohort):

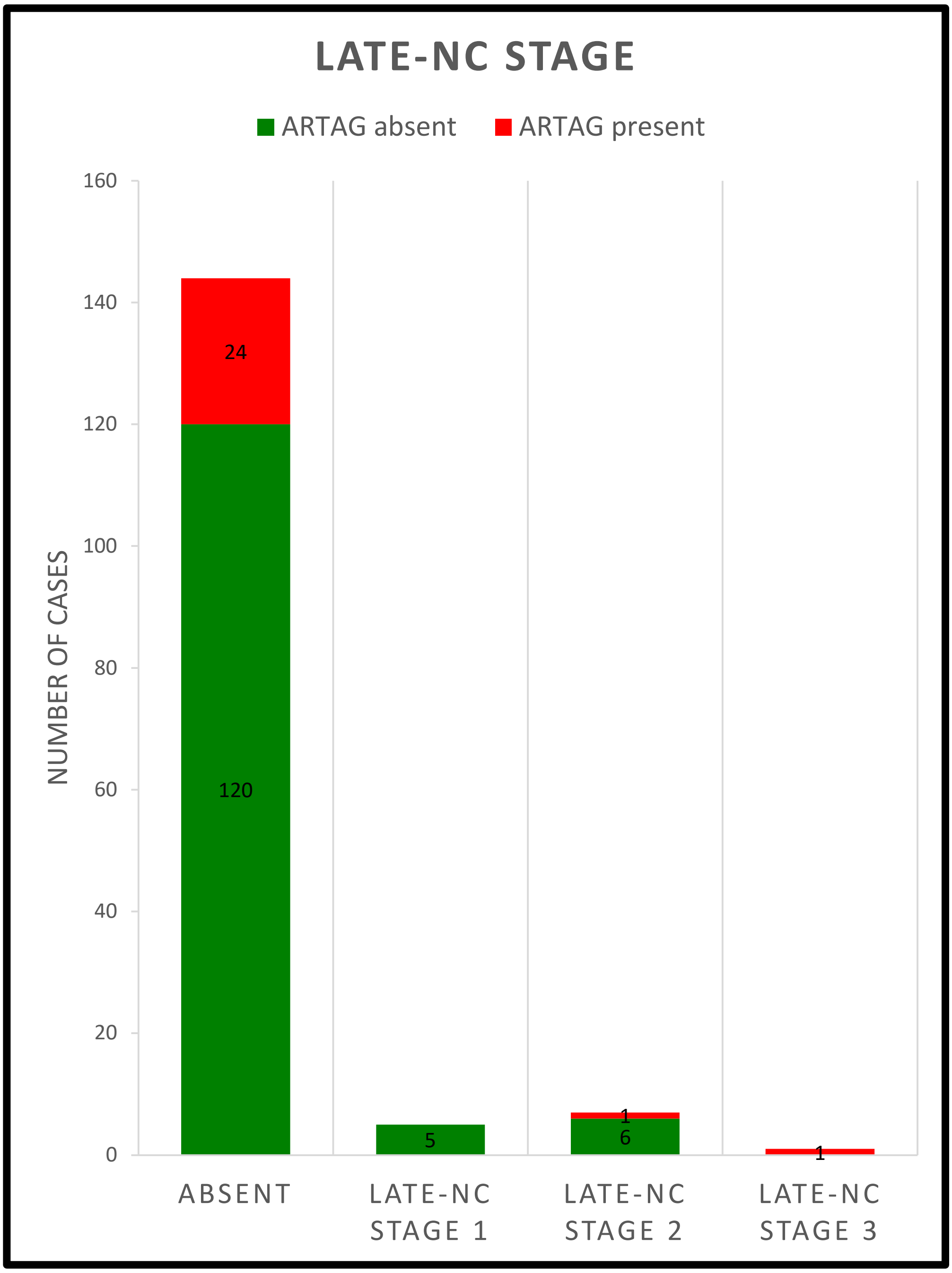

Supplement: awag011_Supplementary_Data [file awag011_supplementary_data.pdf]
